# Effects of Pork Protein Ingestion Prior to and Following Performing the Army Combat Fitness Test on Markers of Catabolism, Inflammation, and Recovery †

**DOI:** 10.3390/nu17121995

**Published:** 2025-06-13

**Authors:** Drew E. Gonzalez, Kelly E. Hines, Ryan J. Sowinski, Landry Estes, Sarah E. Johnson, Jisun Chun, Hudson Lee, Sheyla Leon, Adriana Gil, Joungbo Ko, Jacob Broeckel, Nicholas D. Barringer, Christopher J. Rasmussen, Richard B. Kreider

**Affiliations:** 1 Exercise & Sport Nutrition Laboratory, Department of Kinesiology and Sport Management, Texas A&M University, College Station, TX 77843, USA, , , , , , , , , , , crasmussen@tamu.edu (C.J.R.); 2 Lionel University, Carpinteria, CA 93013, USA; nbarringer@lionel.edu

**Keywords:** essential amino acids, creatine, protein quality, vegetarian diet, exercise

## Abstract

Tactical athletes and military personnel engaged in intense exercise need to consume enough quality protein in their diet to maintain protein balance and promote recovery. Plant-based protein sources contain fewer essential amino acids (EAAs), while pork loin contains a higher concentration of EAAs and creatine than most other animal protein sources. This study aimed to determine whether the ingestion of plant-based or pork-based military-style meals ready-to-eat (MREs) affects recovery from and subsequent Army Combat Fitness Test (ACFT) performance. **Methods:** Twenty-three (*n* = 23) University Corps of Cadets members participated in a randomized, double-blind, placebo-controlled, and crossover-designed study. Diets were prepared by a dietitian, food scientist, and chef to have similar taste, appearance, texture, and macronutrient content. The chef also labeled the meals for double-blind administration. Participants refrained from intense exercise for 48 h before reporting to the lab in a fasted condition with a 24 h urine sample. Participants donated a blood sample, completed questionnaires and cognitive function tests, and consumed a pre-exercise meal. After four hours, participants performed the ACFT according to military standards. Participants were fed three MREs daily while returning to the lab in a fasted condition at 0600 with 24 h urine samples after 24, 48, and 72 h of recovery. On day 3, participants repeated the ACFT four hours after consuming an MRE for breakfast. Participants resumed normal training and returned to the lab after 2–3 weeks to repeat the experiment while consuming the alternate diet. Data were analyzed using general linear model statistics with repeated measures and percent changes from baseline with 95% confidence intervals. **Results:** Results revealed that 3 days were sufficient for participants to replicate ACFT performance. However, those consuming the pork-based diet experienced less muscle soreness, urinary urea excretion, cortisol, inflammation, and depression scores while experiencing a higher testosterone/cortisol ratio and appetite satisfaction. There was also evidence of more favorable changes in red and white blood cells. Conversely, blood lipid profiles were more favorably changed when following a plant-based diet. **Conclusions:** These findings suggest that protein quality and the availability of creatine in the diet can affect recovery from intense military-style exercise. Minimally, plant-based MREs should include 6–10 g/d of EAA and 2–3 g/d of creatine monohydrate to offset dietary deficiencies, particularly in military personnel following a vegetarian diet. Registered clinical trial #ISRCTN47322504.

## 1. Introduction

The International Society of Sports Nutrition (ISSN) recommends that athletes and tactical/occupational personnel involved in heavy training consume 1.4–2.0 g/kg/d of quality protein, ideally in 4–6 meals/snacks per day, containing at least 6 g of essential amino acids (EAAs) with 2–3 g of leucine to optimize protein synthesis for growth and recovery [1,2]. Additionally, individuals should consume at least 2–3 g/d of dietary creatine to maintain muscle and brain creatine levels [3]. However, animal and plant protein sources can differ in EAA, leucine, and creatine content [4]. Plant-based protein sources typically have 30–40% less EAAs than animal protein sources and only trace amounts of creatine [5]. For this reason, it is more difficult for athletes and military personnel to consume enough plant-based protein in their diet to meet daily protein and creatine needs.

Military Ready-to-Eat meals (MRE™) provide ≈1300 calories per meal, containing 170 g (50%) of carbohydrates, 45 g (15%) of protein, and 50 g (35%) of fat [6]. The 2023 MRE meal plans provide 14 menus with primarily animal-based protein (e.g., beef, chicken, sausage, pork, tuna, and cheese) and 9 menus that primarily use plant-based proteins (e.g., beans, rice, and nuts) as the source of protein in the meals [7]. Only one 2023 MRE meal menu uses pork as the source of protein [7]. Research has shown a more rapid digestion, a greater increase in plasma EAAs, and a more pronounced stimulation of muscle protein synthesis following the consumption of protein sources with higher EAA content [1]. Moreover, individuals who consume plant-based diets have lower muscle creatine content, which can limit performance [3]. Consequently, an athlete or active-duty soldier involved in heavy/intense training or combat would potentially have to consume 30–40% more plant-based protein to provide equivalent amounts of EAAs and leucine than animal sources of protein [5], as well as supplement their diet with 2–3 g/d of creatine monohydrate to meet daily needs. Research has indicated that ingesting protein with high EAA content and/or EAA before [8,9], during [10], and/or following intense exercise [11,12] lessens markers of catabolism, inflammation, oxidative stress, immune stress, muscle enzyme efflux/damage, and perceptions of muscle soreness and fatigue, thereby improving the ability to perform a subsequent bout of intense exercise [1,2].

Lean pork is a complete protein with a higher Digestible Indispensable Amino Acid Score (i.e., 117) and concentration of EAAs, leucine, and creatine compared to most other animal and plant-based proteins used in MREs [13,14]. Recent research indicates that ingesting pork protein promotes a more significant increase in muscle protein synthesis and/or a better net protein balance compared to other sources of protein (e.g., egg, tofu, kidney beans, black beans, peanut butter, almonds, and mixed nuts) [14,15], particularly when ingested in per-ounce or -gram equivalent servings as is provided in MREs [14,15]. Theoretically, the use of lean pork as the source of protein in MRE meals before and after performing intense exercise may increase the availability of EAAs and creatine, thereby reducing markers of catabolism, inflammation, oxidative stress, immune stress, muscle enzyme efflux/damage, and perceptions of fatigue and muscle soreness and improve the ability to perform a subsequent bout of intense exercise compared to consuming plant-based proteins used in MREs.

This study aimed to determine if ingesting US Military-designed MRE menu meals [7], containing either pork or plant-based protein sources, before and following performing the Army Combat Fitness Test (ACFT) will lessen markers of catabolism, inflammation, oxidative stress, muscle damage, and perceptions of muscle soreness and/or improve cognitive function and the ability to perform the ACFT following 3 days of recovery. We hypothesized that using pork as the source of protein would lessen the catabolic effects and perception of fatigue associated with performing the ACFT and thereby improve subsequent physical and cognitive performance.

## 2. Methods

### 2.1. Experimental Design

This study was conducted as a randomized, double-blind, placebo-controlled, crossover, and counterbalanced study. The University Human Protection Institutional Review Board (IRB) approved the study (IRB2024-0211, approved 16 February 2024) following the ethical standards for conducting human participant research described in the Declaration of Helsinki. This clinical trial was registered with the International Standard Randomized Control Number Registry (ISRCTN47322504). The independent variable was the protein source in MRE-style meals. Primary endpoints included markers of catabolism, inflammation, oxidative stress, general immune stress, muscle enzyme efflux/damage, ratings of muscle soreness, cognitive function measures, and performance. Secondary endpoints included whole blood red and white cell counts, standard serum clinical safety panels, diet satisfaction ratings, and the frequency and severity of general side effects.

### 2.2. Participants

Healthy males and females who were members of the Texas A&M Corps of Cadets, Ranger Challenge Team, or Delta Company, comprising US Military Veterans, were recruited to participate in this study. Eligibility criteria included the following: (1) age from 18 to 40 at the time of consent; (2) trained male and female members of the Corps of Cadets or Active Military; (3) medically cleared to participate in Corps of Cadets or Active Military physical training activities; (4) ability to comply with study procedures; (5) agree to only ingest meals provided during the study; (6) agree to refrain from alcohol intake and use of non-steroidal anti-inflammatory (NSAIDS), aspirin, and other over-the-counter pain medications for 48 h before and after the completion of each testing session; and (7) availability to complete the study based on the durations of individual visits and scheduling requirements. Exclusion criteria included: (1) being pregnant, breastfeeding, or wishing to become pregnant during the study; (2) having an orthopedic limitation that would prevent participation in the ACFT; (3) having taken weight loss dietary supplements or medications during the last two weeks; (4) having a history within the previous 12 months of alcohol or substance abuse; (5) participants who are heavy smokers (>1 pack/d within the past three months); (6) having known allergies to pork protein (loin and ham) or plant-based proteins (e.g., soybeans, tempeh, tofu, beans, lentils, peas, jackfruit, mushrooms, and seitan wheat-based protein). All participants provided voluntary and written informed consent to participate in this study in compliance with IRB standards.

**Figure 1:**
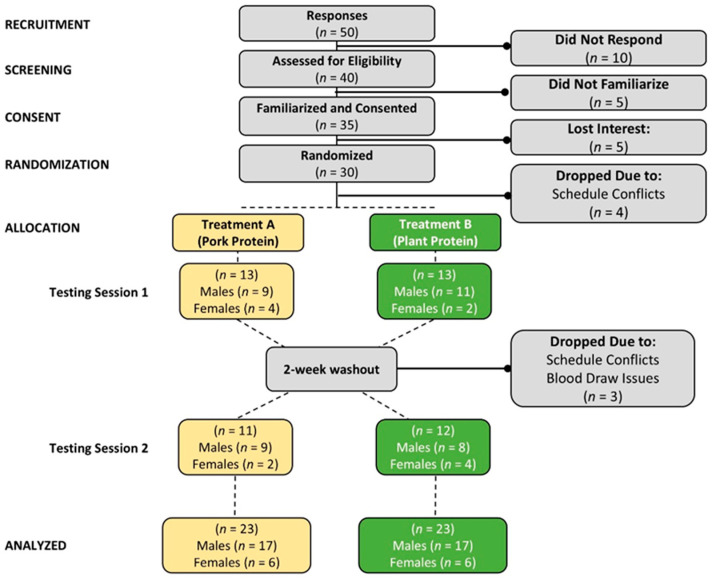
Consolidated Standards of Reporting Trials (CONSORT) diagram of the number of participants screened, consented, randomized, allocated, analyzed, and withdrawals.

Figure 1 shows a Consolidated Standards of Reporting Trials (CONSORT) diagram. A total of 50 individuals responded to the study advertisements, and 40 prospective participants were assessed for eligibility. Of these, 35 volunteers passed the phone screening, were familiarized with the study, and provided informed voluntary consent to participate. A total of 30 participants were randomized into treatments, with 13 individuals (9 males, 4 females) assigned to treatment A (pork-based protein diet) and 13 volunteers (11 males, 2 females) assigned to treatment B (plant-based protein diet), completing testing session one. Participants observed a 2-week washout and then repeated the experiment following the alternate diet intervention. Three participants withdrew from the study due to scheduling conflicts or issues obtaining blood samples. A total of 23 participants completed session two of the study (12 from Treatment A and 11 from Treatment B), including 17 males and 6 females.

### 2.3. Study Timeline

**Figure 2:**
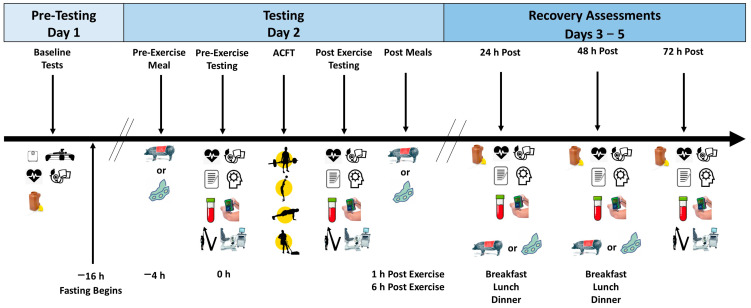
Study session testing sequence. ACFT is the Army Combat Fitness Test.

Figure 2 shows the study timeline and measurements taken during participant visits. Participants were recruited from members of the Texas A&M Corps of Cadets, Ranger Challenge Team, and Delta Company Veterans Outfit for enlisted soldiers pursuing their degrees to become commissioned officers. Prospective participants meeting phone screening eligibility were invited to a familiarization session in which they were informed about the study and provided written informed consent in compliance with the university IRB. Participants completed health history questionnaires; underwent a general physical exam, including the determination of height, weight, and resting hemodynamics (i.e., heart rate and blood pressure); and were informed of the study methods. The participants were also given instructions about how to pick up and when to eat the MRE-style meals and practiced cognitive function tests. Participants were asked to record food and fluid intake for 4 days before testing and replicate this diet before each testing session sequence. Additionally, the participants were asked to refrain from intense exercise training, alcohol intake, and consuming atypical amounts of caffeine and other stimulants not typically consumed in their diet for 48 h before reporting to each testing session. Participants fasted from dinner at about 1700–1800 until reporting to the lab at about 0600 the next day. On each testing session, participants returned 4-day food logs. They were weighed, had their resting heart rate and blood pressure determined, donated a fasting blood sample, and completed questionnaires (i.e., diet satisfaction and side effects). Participants then performed computer-based cognitive function tests and rated muscle soreness using a Visual Analog Scale (VAS) in response to having 50 N of pressure applied using an algometer to three standardized locations on the thighs. Participants were then fed breakfast and returned to the lab four hours later for exercise testing (i.e., the ACFT). Participants performed a standardized warm-up that included static and dynamic stretching and movements. Participants then performed a vertical jump test and the ACFT, including a 3-repetition maximum deadlift, standing medicine ball throw, a 2 min hand release push-up test, plank test, the sprint-drag-carry test, and a 2-mile run. After completing the testing, participants consumed an afternoon meal at about 1300–1400 and a dinner meal around 1900. Participants then fasted before returning to the lab at about 0600 after 24, 48, and 72 h of recovery, where they donated a 24 h urine sample and a blood sample, had their resting heart rate and blood pressure determined, completed questionnaires, performed cognitive function tests, and rated muscle soreness each day of recovery. Participants also repeated the vertical jump and ACFTs on the third recovery day. Participants returned to normal unit activities and were scheduled to repeat the experiment while assigned to the alternate diet intervention after 14–21 days of recovery.

### 2.4. Diet Intervention

The 2023 US-Military MRE meal plans [7] were used as guides to prepare meals using lean pork options for the pork-based diets, while plant protein sources (e.g., soybeans, tempeh, tofu, beans, lentils, peas, jackfruit, mushrooms, and seitan wheat-based protein) were used for the plant-based diets. Meals were prepared by a researcher with a doctorate in Nutrition and Food Sciences and a trained chef in consultation with a registered dietitian (RDN) and a dietetically trained research associate. The chef used plant-based proteins and flavorings that matched the pork protein foods. Participants were aware that we were evaluating whether different dietary proteins affected recovery but were unaware of the protein sources used in the diets. Meals were prepared in a certified metabolic kitchen in the research facility by a trained chef and placed in generic meal containers labeled “A” or “B”. ESHA Food Processor (Version 8.6) Nutritional Analysis software (ESHA Research Inc., Salem, OR, USA) was used to ensure meals met MRE energy and macronutrient recommendations. Food was prepared, packaged, and refrigerated for daily pick-up when returning to the lab for testing. The macro- and micronutrient content, including the protein and creatine content of the meals, are shown in Table 1.

### 2.5. Procedures

#### 2.5.1. Demographics

Body weight and height were determined using a Health-O-Meter Professional 500KL (Pelstar LLC, Alsip, IL, USA) self-calibrating digital scale (±0.02 kg), with values rounded to the nearest tenth. Resting heart rate and blood pressure were measured using a calibrated Connex^®^ ProBP^™^ 3400 digital blood pressure device (Welch Allyn, Tilburg, NL, USA) using standard procedures.

#### 2.5.2. Exercise Intervention and Performance Testing

##### Vertical Jump Power Test

Using standard procedures, peak power was assessed using a Vertec™ (Jump USA, Sunnyvale, CA, USA) before and after 3 days of recovery using the following formula: peak power (W) = ((61.9 × jump height(cm)) + (36 × body mass) + 1822) [16]. Vertical jump distance was obtained from a standing position, with the distance determined from the participant’s farthest vertical reach (with their feet firmly on the ground) to the height reached after jumping vertically. Participants were given three attempts at performing the vertical jump, and the highest jump value was used. Vertical jump testing has been shown to correlate with maximal strength and sprint performance [17].

##### Army Combat Fitness Test

The ACFT is a general physical fitness test used to evaluate a soldier’s physical fitness and readiness [18]. Results are normalized to age and gender and scored, and testing is competitive [18]. The ACFT is considered by military personnel to be physically and mentally challenging and competitive, and it may promote physical and mental fatigue. It is also a standard means to assess soldier readiness [18]. The ACFT included the following: (1) The 3-repetition maximum deadlift (MDL), which assesses the muscular strength fitness component by measuring a soldier’s lower body, grip, and core muscular strength. It requires well-conditioned back and leg musculature and helps soldiers avoid hip, knee, and lower back injuries. Flexibility and balance are secondary fitness components assessed by the MDL. (2) The Standing Power Throw Test (SPT) assesses the power fitness component by measuring a soldier’s ability to generate quick, explosive movements with their upper and lower body. Secondary fitness components assessed by the SPT include balance, coordination, and flexibility. (3) The Hand Release Push-Up–Arm Extension Test (HRP) assesses the muscular endurance fitness component by measuring a soldier’s upper body endurance. The HRP is a strong driver for upper-body and core strength training. Flexibility is a secondary fitness component assessed by the HRP. (4) The Sprint-Drag-Carry Test (SDC) assesses the muscular endurance, muscular strength, anaerobic power, and anaerobic endurance fitness components by measuring a soldier’s ability to sustain moderate to high-intensity muscular work over a short duration. Secondary fitness components assessed by the SDC include balance, coordination, agility, flexibility, and reaction time. (5) The Plank test (PLK) assesses the muscular endurance fitness component by measuring a soldier’s core strength and endurance. Balance is a secondary fitness component assessed by the PLK. (6) The Two-Mile Run (2MR) assesses the aerobic endurance fitness component. Higher aerobic endurance allows a soldier to work for longer and recover more quickly when executing repetitive physical tasks. Tests were performed according to U.S. Military and Corps of Cadets standards. We used this test to promote fatigue and muscle soreness and assess recovery from military training.

### 2.6. Muscle Soreness Assessment

Perception of muscle soreness was evaluated by applying standardized pressure (50 N) using a handheld Commander Algometer (JTECH Medical, Salt Lake City, UT, USA) to three specified areas of the quadriceps muscle using procedures previously described [19]. Participants were asked to mark along a pain rating VAS to show how much muscle soreness they perceived. Reliability using this method in our lab revealed a mean intraclass correlation of 0.909.

### 2.7. Cognitive Function Assessment

#### 2.7.1. Trail Making Test (TMT)

A TMT assesses executive functions, speed, medication management, and inhibition. Participants were provided with printed TMT sheets and asked to connect the links quickly. The time taken to complete the TMT was recorded by a research assistant and used as the score. The TMT is often used in cognitive function testing and is viewed as a reliable executive function test.

#### 2.7.2. Psychomotor Vigilance Task Test (PVTT)

Computerized Mental Performance Assessment (COMPASS) software version 6.0 (Northumbria University, Newcastle, UK) was used to administer the PVTT. This test assesses sustained attention reaction times through responses to visual light stimuli, requiring participants to press a keyboard button in response to a randomly illuminating light on the screen every few seconds. The number of times the button was not pressed and the speed of response were measured, with sleepiness quantified as the number of lapses in attention during the test. This test has been used routinely in cognitive function testing, including in the military.

#### 2.7.3. Profile of Mood States (POMS) Inventory

The shortened version of the POMS was used to assess tension-anxiety, depression-dejection, anger-hostility, fatigue-inertia, confusion-bewilderment, and total mood disturbance. This is a reliable and standard test used among athletes and the military to assess vigor and fatigue.

### 2.8. Blood Collection and Analysis

Fasting whole blood samples were collected using standard phlebotomy procedures by certified phlebotomists into two 7.5 mL BD Vacutainer serum separation tubes (SSTs) and one 3.5 mL BD Vacutainer ethylenediaminetetraacetic acid (EDTA) tube (Becton, Dickinson and Company, Franklin Lakes, NJ, USA). The SSTs were left at room temperature for about 15 min to allow clotting, then centrifuged at 3000× *g* for 10 min in a refrigerated (4 °C) Thermo Scientific Heracus MegaFuge 40R centrifuge (Thermo Electron North America LLC, West Palm Beach, FL, USA). Serum from one SST was aliquoted into polypropylene Eppendorf microcentrifuge tubes (Eppendorf, Enfield, CT, USA) and stored at −80 °C until analyzed. The remaining SST and EDTA tubes were sent to the Clinical Pathology Laboratory in Bryan, TX, USA, for a complete blood count with differentiation (red blood cells, white blood cells, hemoglobin, hematocrit, mean corpuscular volume, mean corpuscular hemoglobin, mean corpuscular hemoglobin concentration, red cell distribution width, neutrophils, lymphocytes, monocytes, eosinophils, basophils, and immature granulocytes) and a comprehensive metabolic panel (glucose, blood urea nitrogen, creatinine, glomerular filtration rate, sodium, potassium, chloride, carbon dioxide, protein, globulin, bilirubin, alkaline phosphatase, aspartate transferase, alanine transferase, total cholesterol, triglycerides, high-density lipoprotein cholesterol, low-density lipoprotein cholesterol, very low-density lipoprotein cholesterol, low-density lipoprotein cholesterol/high-density lipoprotein cholesterol risk ratio, and total cholesterol/high-density lipoprotein cholesterol risk ratio).

Serum cortisol and testosterone concentrations were determined using commercially available enzyme-linked immunoassay (ELISA) kits (Alpco Diagnostics, Salem, NH, USA, 11-CRLHU-E01, 11-TESHU-E01) and a BioTek Epoch 2 plate reader (BioTek Instruments, Winooski, VT, USA) with BioTek Gen 5 software, according to manufacturer instructions. The manufacturer reported that quality control analysis on the kit lot numbers used in this study showed a variability of 5% for cortisol and 6% for testosterone on low to high calibrators with an acceptable range of 1.2–1.4% binding, respectively. Serum cytokines, including interleukin (IL) −1β, 2, 4, 5, 6, 8, and 10; granulocyte-macrophage colony-stimulating factor (GM CSF); interferon-γ (IFN-γ); and tumor necrosis factor-α (TNF-α) were measured with a commercially available Cytokine Human Magnetic 10-plex Panel on a Luminex 200 Instrument System and a Milliplex Analyzer (ThermoFisher Scientific, Vienna, Austria) using xPONENT^TM^ software version 4.3, following company instructions. Inter- and intra-assay CV calculations from previous experiments in our lab yielded 2.2–17.5% and 3.3–9.8%, respectively.

### 2.9. Urine Collection and Analysis

Twenty-four-hour urine samples were collected the day before and for 3 days after the experimental testing session. Urine samples were vortexed, stored in urine collection tubes with preservatives, and transported to the Clinical Pathology Laboratory in Bryan, TX, USA, for nitrogen and creatinine analysis using standard procedures.

### 2.10. Questionnaires

#### 2.10.1. Readiness to Perform Questionnaire

Participants rated how well they slept the night before testing, whether they were looking forward to the workout/exercise activity (i.e., ACFT), how optimistic they were about their performance, how vigorous and energetic they felt, their appetite level, and the amount of muscle soreness they perceived on a Readiness to Perform questionnaire using the following scale: 1 (strongly disagree), 2 (disagree), 3 (neutral), 4 (agree), and 5 (strongly agree). This test has been used in several studies from our lab to assess performance readiness and recovery.

#### 2.10.2. Diet Satisfaction Questionnaire

Participants were asked to rank the frequency and severity of the symptoms (i.e., hypoglycemia, dizziness, headache, fatigue, and stomach upset) and side effects (i.e., dizziness, headache, tachycardia, heart skipping/palpitations, shortness of breath, nervousness, blurred vision, and any other adverse effects). The test–retest reliability of this assessment showed a coefficient variation (CV) of 1.2–2.6 with single-item survey intraclass correlations between 0.6 and 0.88.

### 2.11. Statistical Analysis

Data were analyzed using IBM^®^ Version 29 SPSS^®^ statistical analysis software (IBM Corp., Armonk, NY, USA). A crossover sample size of 25 was selected based on our prior work showing that crossover and parallel study designs, 12 participants per treatment, were sufficient to detect statistically significant differences in markers of catabolism, inflammation, oxidative stress, muscle soreness, and cognitive function measures with various nutritional interventions [19,20,21,22,23,24,25]. We also considered the reported effect sizes in the related literature. We used the reported means, standard deviations, and statistically significant mean differences reported in these studies to calculate power, assuming an 80% power with a 5–10% standard deviation to the mean and a 5–10% improvement in primary outcomes. We used general linear model (GLM) multivariate and univariate analyses with repeated measures of time and treatments to assess primary and secondary outcomes. Sphericity was assessed using Mauchly’s test, while skewness and kurtosis statistics assessed normality. The Wilks’ Lambda and Greenhouse–Geisser univariate correction tests were used to assess time and treatment × time interaction effects to adjust for F-value inflation if the assumption of sphericity was violated. Fisher’s Least Significant Difference tests and 95% upper and lower confidence intervals (CIs) at pre-planned contrasts of interest were used to assess pairwise comparisons of the means and post hoc tests. Additional tests were not employed to correct for multiple comparisons since the participants served as their control (i.e., repeated × repeated design), and the Greenhouse–Geisser correction was used to adjust for F-value inflation [26,27]. The type I error (*p*-level) probability was 0.05 or less, while statistical trends were noted when *p*-values ranged between >0.05 to <0.10. The clinical significance of the findings was evaluated by assessing mean changes or percentage mean changes from baseline with 95% confidence intervals (CIs) [28]. Means and 95% CIs above or below baseline or PLA were considered statistically and clinically significant [28]. We did not use measurement error or consider clinician perspectives regarding minimally meaningful clinical changes to assess clinical significance. However, test–retest reliability CVs are reported for readers to consider. Data are presented as means ± standard deviations (SD) or mean percent changes from baseline (mean change [LL, UL]). Partial Eta squared *(ηₚ^2^*) values were used to assess effect size, with values of 0.01 (small effect), 0.06 (medium effect), and 0.14 (large effect) [29]. Categorical questionnaire data were analyzed using a Chi-squared analysis. Missing data were replaced using the series means for numerical data [30], while responses to categorical survey questions (i.e., ordinal data) were replaced using the most frequent response or value method [31]. Both methods have been reported to be appropriate and valid for replacing missing data [30,31]. Our statistical approach provides a comprehensive statistical analysis that goes beyond assessing statistical differences using *p*-levels by reporting the effect sizes and pairwise comparisons of contrasts of interest to reduce the likelihood of type II statistical error and help researchers decide whether additional research with larger populations is warranted [32,33,34] and the clinical significance of findings by assessing percent changes from baseline with 95% CIs [28,32,33,34,35,36,37].

## 3. Results

### 3.1. Demographic Data

Table S1 displays the participant demographic data. Participants were 20.0 ± 2.1 years old, 173.1 ± 8.5 cm tall, weighed 74.9 ± 10.8 kg, had a body mass index (BMI) of 24.9 ± 2.6 kg/m^2^, had a resting heart rate of 63.3 ± 11.5 bpm, had a resting systolic blood pressure of 113.9 ± 9.4 mmHg, and had a resting diastolic blood pressure of 68.6 ± 6.7 mmHg. No significant differences in the demographic variables were observed between treatment sessions. Sex differences (*p* < 0.001) were observed in all baseline measures except age (*p* = 0.351), BMI (*p* = 0.219), and resting diastolic blood pressure (*p* = 0.089). Changes in anthropometric and resting hemodynamic variables are displayed in Table S2. No significant treatment × time effects were observed in weight, BMI, resting heart rate, or resting blood pressure between treatment sessions after 24, 48, or 72 h of recovery. A time effect was observed in weight and BMI from baseline after 48 h, but these changes were small and not observed after 72 h, with no differences between treatments.

### 3.2. Performance

**Figure 3:**
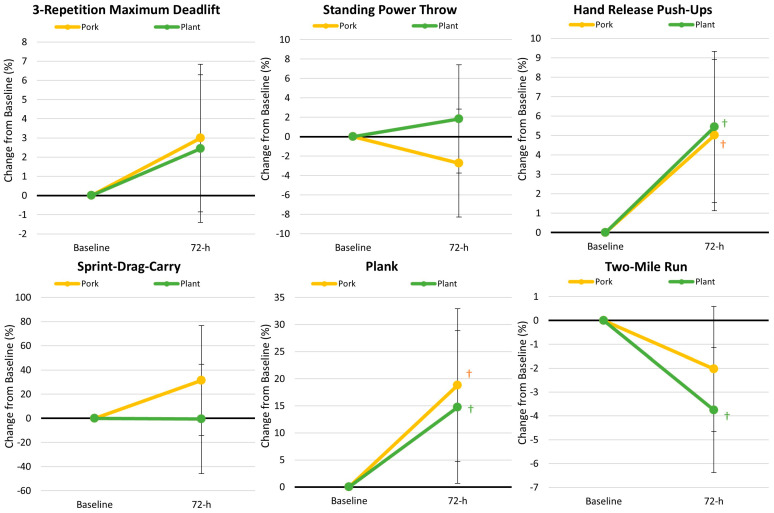
Army Combat Fitness Test results. Data are displayed as the mean changes from baseline with 95% confidence intervals. † = *p* < 0.05 from baseline values.

Table S3 presents performance-related data. The overall GLM analysis revealed a significant time effect (*p* < 0.001, $\eta_{p}^{2}$ = 0.463, large effect), while no significant interaction effects were observed between treatments (*p* = 0.811, $\eta_{p}^{2}$ = 0.088, moderate effect). Univariate analysis revealed significant time effects on the hand release push-up, plank test, and two-mile run, with 72 h recovery values being significantly better than baseline values. No differences were observed between treatments. Figure 3 shows the analysis of percentage changes in the ACFTs performed. Participants improved hand-release push-up repetitions (Pork 5.0% [1.1, 8.9], *p* = 0.013; Plant 5.4% [1.5, 9.3], *p* = 0.007) and plank test time from baseline (Pork 18.8% [4.7, 32.9], *p* = 0.010; Plant 14.8% [0.6, 28.9], *p* = 0.041) with no significant difference observed between diet treatments. Two-mile run times were significantly faster with the plant-based protein diet (−3.8% [−6.4, −1.1], *p* = 0.006) while non-significantly decreased with the pork protein diet treatment (−2.0% [−4.7, 0.6], *p* = 0.125). Vertical jump performance did not change significantly over time. Table S4 presents the ACFT percentile ranking scores. Hand release push-up repetitions (Pork 1.7% [0.4, 3.0], *p* = 0.011; Plant 0.9% [−0.4, 2.2], *p* = 0.158) and total ACFT score values (Pork 22.9 [5.9, 39.9], *p* = 0.009; Plant 12.9 [−4.1, 29.9], *p* = 0.134) significantly increased from baseline with pork diet treatment, while two-mile run times increased from baseline with plant diet treatment (Pork 2.4% [−0.4, 5.2], *p* = 0.087; Plant 3.8% [1.0, 6.6], *p* = 0.009) with no significant differences observed between diet treatment interventions.

### 3.3. Perceptions of Muscle Soreness

**Figure 4:**
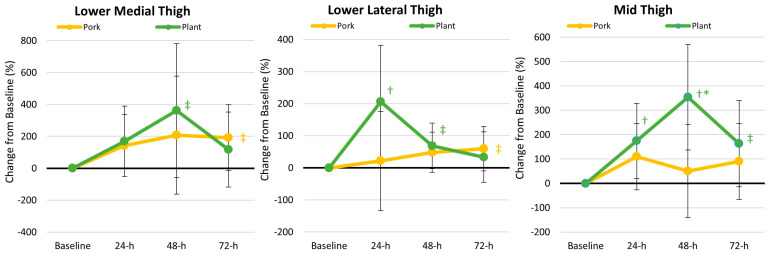
Changes in perceptions of muscle soreness. Data are displayed as mean changes from baseline with 95% confidence intervals. † = *p* < 0.05 (‡ = *p* > 0.05 to *p* < 0.10) from baseline values. * = *p* < 0.05 difference between diet treatments.

Table S5 presents the participants’ muscle soreness ratings in response to performing the ACFT. Multivariate Wilk’s Lambda identified a significant time (*p* = 0.027, *ηₚ*^2^ = 0.047, small effect) with no treatment × time effect (*p* = 0.553, *ηₚ*^2^ = 0.020, small effect). Univariate analysis indicated that participants perceived an increase in pain in the lower medial (*p* = 0.007, *η_p_*^2^ = 0.090, moderate effect) and lower lateral thigh (*p* = 0.040, *ηₚ*^2^ = 0.065, moderate effect) locations with no effect on mid-thigh pain perception. Percentage changes in pain ratings were generally higher in the plant-based diet (see Figure 4), indicating that participants experienced less muscle soreness after performing the ACFT when consuming a pork protein diet than a plant protein diet.

### 3.4. Cognitive Function Assessment Results

#### 3.4.1. Trail Making Test (TMT)

**Figure 5:**
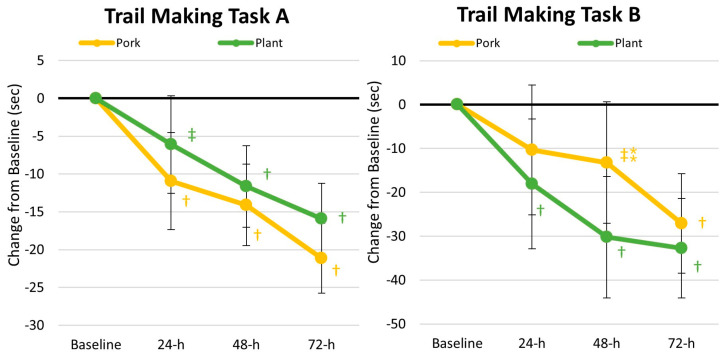
Changes in trail making task performance. Data are displayed as the mean percentage changes from baseline with 95% confidence intervals. † = *p* < 0.05 (‡ = *p* > 0.05 to *p* < 0.10) from baseline values. ⁑ = *p* > 0.05 to *p* < 0.10 difference between diet treatments.

Table S6 presents results from the trail making task test. Multivariate Wilk’s Lambda analysis revealed a significant time (*p* < 0.001, *ηₚ*^2^ = 0.281, large effect) with no treatment × time effect (*p* = 0.330, *ηₚ*^2^ = 0.026, small effect). Univariate analysis indicated a significant improvement in the time required to complete TMT tasks A and B, with no significant interaction effects observed between treatments. Percentage changes in TMT performance are shown in Figure 5. Performance improved with both treatments, and no significant differences were observed.

#### 3.4.2. Psychomotor Vigilance Task Test (PVTT)

**Figure 6:**
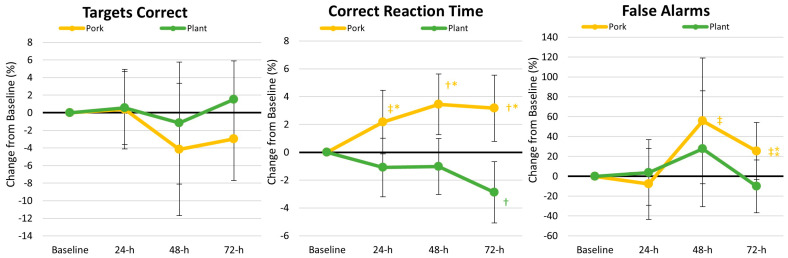
Changes in psychomotor Vigilance Task Test (PVTT) results. Data are displayed as the mean percentage changes from baseline with 95% confidence intervals. † = *p* < 0.05 (‡ = *p* > 0.05 to *p* < 0.10) from baseline values. * = *p* < 0.05 (⁑ = *p* > 0.05 to *p* < 0.10) difference between diet treatments.

Table S7 shows the results from the PVTT. Overall multivariate analysis revealed a non-significant time (*p* = 0.472, *ηₚ*^2^ = 0.022, small effect) with a significant treatment × time effect (*p* = 0.038, *ηₚ*^2^ = 0.044, small effect). Univariate analysis revealed no significant time effects. A significant interaction effect was observed in the correct response reaction time (*p* = 0.002, *ηₚ*^2^ = 0.111, moderate effect). Reaction time for correct responses tended to be slower with plant protein intake (16.3% [−0.2, 32.6], *p* = 0.052). Analysis of percent changes from baseline (Figure 6) revealed that correct reaction time and false alarms were generally higher with pork protein intake.

#### 3.4.3. Readiness to Perform Questionnaire

Table S8 presents the results of participants’ perceptions of readiness to perform. Multivariate analysis revealed a significant time (*p* = 0.002, *ηₚ*^2^ = 0.097, medium effect) with a non-significant treatment × time interaction effect (*p* = 0.148, *ηₚ*^2^ = 0.060, moderate effect). Univariate analysis revealed significant time effects in responses to “I am looking forward to today’s workout” (*p* < 0.044, *ηₚ*^2^ = 0.068, moderate effect) and “I am optimistic about my future performance” (*p* < 0.007, *ηₚ*^2^ = 0.096, moderate effect). A significant interaction effect was observed in “My appetite is great” (*p* < 0.047, *ηₚ*^2^ = 0.061, moderate effect), where values significantly decreased with the plant-based diet. Changes from baseline with 95% CIs are illustrated in Figure S1.

#### 3.4.4. Profile of Mood States (POMS) Inventory

Table S9 presents results from the Profile of Mood States inventory. Multivariate analysis revealed a significant time (*p* < 0.001, *ηₚ*^2^ = 0.128, large effect) with a non-significant treatment × time interaction effect (*p* = 0.927, *ηₚ*^2^ = 0.026, small effect). Univariate analysis revealed a significant decrease over time in vigor ratings (*p* < 0.001, *ηₚ*^2^ = 0.225, large effect) and Total Mood Disturbance Score (TMDS, *p* < 0.001, *ηₚ*^2^ = 0.196, large effect) with no significant treatment × time interaction effect observed. Analysis of percentage changes from baseline (Figure S2) revealed that depression ratings tended to be lower after 48 h in the pork diet treatment.

### 3.5. Blood Analysis

#### 3.5.1. Whole Blood Analysis

Cell blood count (CBC) results expressed as absolute values and percentage differentials are presented in Table S10. No overall time (*p* = 0.183, *ηₚ*^2^ = 0.188, large effect) or treatment × time interaction effects (*p* = 0.294, *ηₚ*^2^ = 0.188, large effect) were observed among CBC data. Univariate analysis revealed no time effects among variables. Mean corpuscular volume values (*p* = 0.072, *ηₚ*^2^ = 0.052, small effect) tended to interact, while basophil values interacted between diets (*p* = 0.018, *ηₚ*^2^ = 0.073, moderate effect). However, changes were small and well within normal values. The neutrophils-to-lymphocytes ratio, a general marker of immune stress, was not significantly different between diet treatments. Percentage changes from baseline in these variables are shown in Figure S3a,b.

#### 3.5.2. Renal Function and Electrolytes

Table S11 shows serum renal function and electrolyte data. The Multivariate Wilk’s Lambda Time (*p* < 0.001, *ηₚ*^2^ = 0.188) effect was observed with no significant treatment × time interaction observed (*p* = 0.833, *ηₚ*^2^ = 0.057). Although some time effects were observed in univariate analysis, no significant treatment × time interaction effects were observed between diet treatments in these markers. All values were within normal ranges for active individuals. Figure S4 presents the mean percentage changes in these variables.

#### 3.5.3. Blood Lipid Analysis

**Figure 7:**
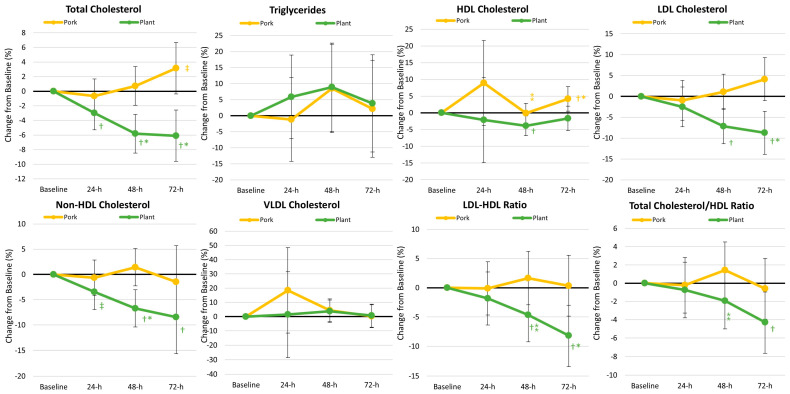
Percentage changes in blood lipid-related variables. Data are displayed as the mean percentage changes from baseline with 95% confidence intervals. † = *p* < 0.05 (‡ = *p* > 0.05 to *p* < 0.10) from baseline values. * = *p* < 0.05 (⁑ = *p* > 0.05 to *p* < 0.10) difference between diet treatments.

Table S12 presents blood lipid data. GLM analysis revealed that lipid levels tended to differ over time (*p* < 0.066, *ηₚ*^2^ = 0.087, moderate effect), while a significant treatment × time interaction was revealed (*p* = 0.013, *ηₚ*^2^ = 0.103, moderate effect). Univariate analysis revealed significant interaction effects in total cholesterol (*p* < 0.001, *ηₚ*^2^ = 0.194, large effect) and low-density lipoprotein cholesterol (*p* < 0.001, *ηₚ*^2^ = 0.170, large effect), while the low-density lipoprotein cholesterol/high-density lipoprotein cholesterol ratio tended to interact (*p* < 0.077, *ηₚ*^2^ = 0.055, small effect). Generally, total cholesterol and low-density lipoprotein cholesterol concentrations were lower following the plant-based diet intervention, although values were low and differences between diet interventions were small. Figure 7 shows the percentage changes observed in blood lipids.

#### 3.5.4. Protein and Enzyme Markers

Markers of protein degradation and muscle and liver enzyme efflux data are presented in Table S13. GLM analysis revealed a time effect (*p* < 0.001, *ηₚ*^2^ = 0.153, moderate effect) with a non-significant treatment × time interaction effect (*p* = 0.642, *ηₚ*^2^ = 0.060, moderate effect). Univariate analysis revealed time effects in albumin (*p* = 0.041, *ηₚ*^2^ = 0.063, moderate effect), total bilirubin (*p* = 0.025, *ηₚ*^2^ = 0.082, moderate effect), alkaline phosphatase (*p* = 0.014, *ηₚ*^2^ = 0.083, moderate effect), aspartate aminotransaminase (*p* = 0.027, *ηₚ*^2^ = 0.082, moderate effect), lactate dehydrogenase (*p* = 0.036, *ηₚ*^2^ = 0.079, moderate effect), and creatine kinase (*p* = 0.006, *ηₚ*^2^ = 0.120, moderate effect). However, no significant interaction effects were observed between diet interventions. Figure S5 presents the mean percentage changes in these variables.

#### 3.5.5. Hormonal Markers of Anabolism and Catabolism

**Figure 8:**
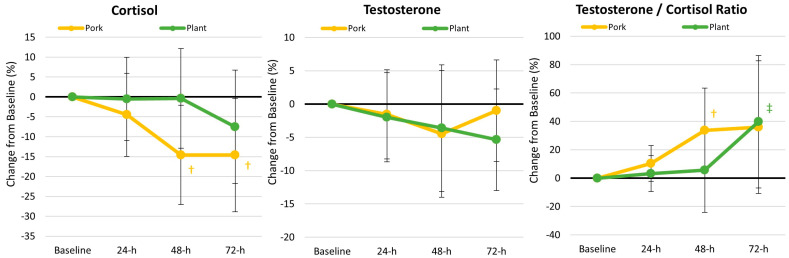
Percent changes in anabolic and catabolic hormones. Data are displayed as the mean percentage changes from baseline with 95% confidence intervals. † = *p* < 0.05 (‡ = *p* > 0.05 to *p* < 0.10) from baseline values.

Table S14 shows data related to cortisol (catabolic hormone) and testosterone (anabolic hormone). Higher cortisol levels indicate greater catabolism and stress, while the ratio of testosterone to cortisol (T/C) is a marker of anabolic/catabolic status. A higher T/C ratio reflects greater anabolism, while a reduction indicates greater catabolism. GLM analysis revealed a time effect (*p* = 0.038, *ηₚ*^2^ = 0.044, small effect) with a non-significant treatment × time interaction effect (*p* = 0.894, *ηₚ*^2^ = 0.011, small effect). Univariate analysis revealed a significant time effect for cortisol (*p* = 0.005, *ηₚ*^2^ = 0.096, moderate effect) with no other time or interaction effects observed among these variables. Analysis of percent mean changes (Figure 8) from baseline revealed that cortisol levels significantly decreased over time after 48 and 72 h in the pork diet treatment, while cortisol levels did not significantly change over time in the plant diet treatment. No changes over time were observed in the percentage change in testosterone. However, the T/C ratio significantly increased above baseline in the pork diet treatment after 48 h, while the T/C ratio only tended to increase from baseline after 72 h. These findings suggest that participants experienced less stress and a more favorable anabolic status when consuming pork as the source of protein compared to a plant-based diet. However, although time effects were observed, the differences were not significantly different at the time points studied.

#### 3.5.6. Markers of Inflammation

Table S15 shows cytokine inflammation-related markers. Pro-inflammatory cytokines include IL-1β, TNF-α, IFN-γ, and GM-CSF, while IL-4 and IL-10 are considered anti-inflammatory cytokines [38]. IL-6 acts as a pro- and anti-inflammatory cytokine. IL-2 is a cytokine that activates white blood cells and immunity, IL-5 produces T-helper cells, IL-8 is a macrophage-derived chemokine that triggers rapid migration of neutrophils, and GM-CSF stimulates the production of neutrophils, eosinophils, and basophils that influence the immune system. GLM analysis revealed a significant overall time (*p* < 0.238, *ηₚ*^2^ = 0.087, moderate effect) or treatment × time effects (*p* = 0.719, *ηₚ*^2^ = 0.063, moderate effect) were observed. No significant time or interaction univariate effects were observed. Figure S6 shows the percentage change in cytokine markers. IL-1β and IL-2 increased from baseline with the plant-based diet, GM-CSF and IL-5 increased with the pork-based diet, and IL-8 levels tended to decrease. IL-2 values were significantly lower with the plant-based diet after 48 h. No other differences were observed between diet treatments.

### 3.6. Urinary Markers

**Figure 9:**
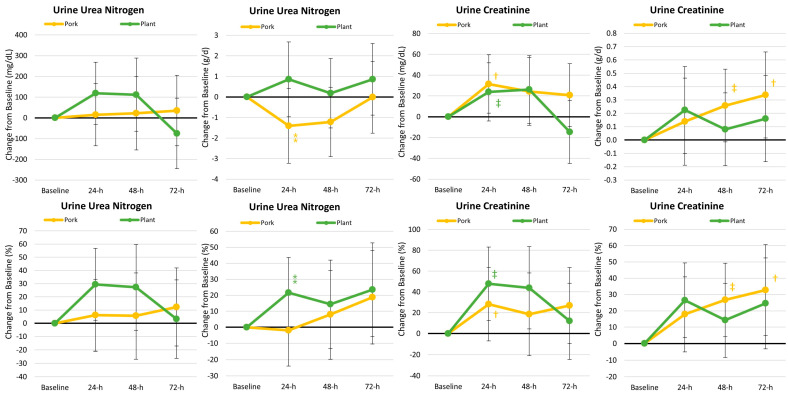
Mean and percentage changes in urinary urea nitrogen and creatinine. Data are displayed as the mean percentage changes from baseline with 95% confidence intervals. † = *p* < 0.05 (‡ = *p* > 0.05 to *p* < 0.10) from baseline values. ⁑ = *p* > 0.05 to *p* < 0.10 difference between diet treatments.

Table S16 shows the urinary markers of catabolism. Overall time (*p* < 0.001, *ηₚ*^2^ = 0.085, moderate effect) and treatment × time effects (*p* = 0.012, *ηₚ*^2^ = 0.063, moderate effect) were observed, indicating urinary urea nitrogen and creatinine differed between groups. Univariate analysis revealed a significant time effect for urine creatinine expressed in mg/dL (*p* = 0.007, *ηₚ*^2^ = 0.090, moderate effect). Figure 9 shows the mean and percentage changes from the baseline in these markers. Participants consuming pork-based MREs experienced less urine nitrogen excretion, indicative of less protein catabolism.

### 3.7. Questionnaires

#### 3.7.1. Diet Satisfaction Questionnaire

Table S17 presents diet satisfaction-related questions. GLM analysis revealed a significant time (*p* < 0.015, *ηₚ*^2^ = 0.072, moderate effect) but no treatment × time effects (*p* = 0.409, *ηₚ*^2^ = 0.039, small effect). Univariate analysis identified significant time effects in ratings of appetite satisfaction (*p* = 0.011, *ηₚ*^2^ = 0.086, moderate effect) and hunger (*p* = 0.009, *ηₚ*^2^ = 0.086, moderate effect) and a tendency toward significance in hunger ratings (*p* = 0.053, *ηₚ*^2^ = 0.057, small effect).

#### 3.7.2. Self-Reported Side Effects

Table S18 presents the frequency and severity of side effects monitored. Chi-squared analysis revealed no significant differences in ratings of the frequency or severity of dizziness, headache, tachycardia, heart palpitations, dyspnea, nervousness, blurred vision, or other side effects. Participants rated most as none or minimal.

## 4. Discussion

The purpose of the study was to determine if consuming MRE menu meals [7], containing pork or plant-based protein before and after the ACFT, affects markers of catabolism, inflammation, oxidative stress, muscle damage, and perceptions of muscle soreness and/or improves cognitive function and the ability to perform the ACFT following 3 days of recovery. The main findings of this study were that consuming MREs containing pork decreased perceptions of muscle soreness, urinary markers of catabolism, cortisol, inflammatory markers, and depression scores while promoting a higher testosterone/cortisol ratio and appetite satisfaction. However, the performance of ACFT tasks was not affected. The following provides a more detailed discussion about the results of primary and secondary variables, limitations, and recommendations.

### 4.1. Primary Outcomes

#### 4.1.1. Markers of Performance, Degradation, and Recovery

Military MREs provide about 1300 kcals/meal, containing about 170 g (50%) of carbohydrates, 45 g (15%) of protein, and 50 g (35%) of fat [6]. Consuming three MREs per day would provide about 3900 kcals/day, consisting of 6.4 g/kg/d of carbohydrates, 1.7 g/kg/d of protein, and 1.9 g/kg/d of fat for the average-sized army ranger. While this seemingly would provide enough protein to meet energy needs, energy expenditure among active-duty soldiers has been reported to range from 2342 to 7122 kcals/day [2]. Consequently, warfighters often endure physiological, psychological, and environmental stressors during training and combat operations that create an energy deficit, resulting in increased catabolism of whole-body and skeletal muscle protein, muscle loss, impaired recovery, and/or poor performance. For this reason, it is paramount to consider methods to deliver not only a greater amount of protein but also high-quality protein to minimize the deleterious effects of military-specific occupational demands.

In this study, we found evidence that the pork-based MRE meals promoted better recovery, as shown by the reduced perceptions of muscle soreness, lower physiological stress hormone responses, greater anabolic status, and less/blunted inflammatory response. Regarding perceptions of muscle soreness, following the consumption of the plant-based MRE meal, the cadets displayed elevated perceptions of muscle soreness; more specifically, the cadets experienced increased perceptions of mid-thigh muscle soreness 24 h (175%), 48 h (354%), and 72 h (163%), following the completion of the ACFT. In contrast, the cadets did not display the same outcome when consuming pork-based MRE meals. Furthermore, perceptions of muscle soreness for the lower medial thigh (Plant at 48 h: 362%; Pork at 72 h: 192%) and lower lateral thigh (Plant at 24 h: 206%, 48 h: 69%; Pork at 72 h: 60%) were generally higher following the plant-based MRE meal regimen. These findings contrast with prior studies that have evaluated whether different types of protein affect perceptions of muscle soreness after exercise [39,40]. For example, Saracino and colleagues [41] had 27 recreationally active, middle-aged men follow a standardized diet (55% carbohydrate, 15% protein, and 30% fat) for five days before administering a 40 g bolus of whey hydrolysate, whey isolate, or a combination of rice and pea protein before sleep. The following day, participants performed five sets of 15 repetitions of maximal eccentric voluntary contractions on the knee extensors and flexors. The researchers reported that muscle soreness increased following the exercise bout (*p* < 0.001), but neither protein regimen affected perceptions of muscle soreness. Kritikos et al. [39] reported that increasing dietary protein intake to 1.5 g/kg/d using whey or soy protein supplements for one week before intense exercise did not differentially affect the deterioration in performance or affect perceptions of muscle soreness in soccer players. Nieman and coworkers [40] reported that dietary supplementation of 0.9 g/kg/d of pea protein or whey protein (2.15 g/kg/d) had no effects on perceptions of muscle soreness after eccentric exercise in untrained individuals compared to a water control. Finally, Xia and associates [42] reported that 25 g of oat protein for 14 days before and 4 days following downhill running attenuated perceptions of muscle soreness and markers of catabolism in untrained individuals. Differences in these studies may be due to the dietary control, types of exercise employed, and/or the training status among studies.

Regarding physiological stress, anabolic status, and inflammatory biomarkers, we found that the pork-based MRE meals outperformed the plant-based MRE meals. For instance, a significant interaction effect (*p* = 0.012, *ηₚ*^2^ = 0.063) was observed in urinary urea nitrogen and creatinine concentrations, indicative of less protein degradation with pork-based MREs. Additionally, serum cortisol concentrations decreased from baseline to 48 h (−14.5%) and 72 h (−14.6%) following the pork-based meal, with an increased testosterone/cortisol ratio (33.6%) at 48 h post ACFT. Furthermore, cadets consuming the plant-based MRE meal experienced an increase in IL-1β (6.1%), whereas those consuming the pork-based meals had elevated IL-5 levels (6.2%) and reduced IL-8 levels (−8.8%) at the 48 h mark. Previous reports have shown that tactical-specific operations/conditions or training can result in elevated stress and inflammatory biomarkers [43,44]. For instance, Hamarsland et al. [45] assessed the inflammatory and muscle damage biomarker response to a so-called “hell week” intervention, consisting of sleep deprivation, caloric restriction, and extreme physical exertion for 20 h a day. The research team found that immediately after the military stressor, participants experienced a 154% increase in cortisol concentrations, with concentrations remaining elevated at 24 h (63%) and up to the 72 h (58%) mark [45]. McClung and colleagues [46] also demonstrated that Norwegian soldiers participating in a 54 km ski march winter training exercise experienced a 59% increase in IL-6 from pre-march concentrations. To our knowledge, this is the first study to assess physiological stress and inflammatory biomarkers pre- and post-ACFT. Considering these objective biomarkers alongside the subjective perceptions of muscle soreness, our results suggest that the pork-based diet may reduce physiological stress and inflammatory responses to strenuous military-specific training while enhancing the perception of soreness, ultimately promoting faster recovery.

#### 4.1.2. Markers of Cognition

Without question, adequate energy and macronutrient provisions are critical for promoting optimal health, performance, recovery, and readiness of military personnel, and recent interest has been garnered in the influence of nutrition on cognitive health and function [2,47]. In addition, military personnel can experience challenges in their sleep schedule (resulting in sleep loss and deprivation) and compromise their mood and mental well-being. Reserve Officer Training Corps (ROTCs) and Corps of Cadets are not immune to these challenges; in fact, cadets often have to navigate physically and mentally demanding conditions (balancing college academics and extracurriculars), which can pose a risk to their health [48]. Ultimately, the stressors encountered by military personnel/cadets can negatively impact cognitive function, including attention, working memory, and executive function. Therefore, efforts to boost cognitive resilience (i.e., the degree to which cognitive function can withstand stress) are paramount for military personnel and cadets alike. To our knowledge, our study is the first to assess cognitive function before and after the ACFT. We found evidence that TMT performance improved following the consumption of both the plant- and pork-based meals, demonstrating an improvement in one’s executive function and cognitive flexibility over the 3 days of recovery (see Figure 5). Furthermore, the plant-based diet led to better PVTT correct reaction times and false alarms than the pork-based diet (see Figure 6). Interestingly, previous reports have suggested that plant-based proteins may be favorably associated with cognitive function and reduced risk of cognitive decline compared to high animal-based protein diets [49]. However, it is currently difficult to conclude whether a specific dietary pattern is more advantageous than another within military personnel, as Teo and colleagues [50] conducted a systematic review of whole dietary patterns to optimize cognition for military readiness and found that five of the six acceptable studies demonstrated conflicting results across the outcome variables. Our findings appear to corroborate those of Teo et al. [50] in that we identified mixed results, demonstrating that both protein regimens improve cognitive function. Further research is warranted to better elucidate these findings and the impact of the protein source on cognitive function, especially following tactical/military-specific stressors.

There are additional cognitive well-being and psychological factors that can be affected by tactical- or military-specific stressors, such as mood, readiness for performance, and sleep. We found that those consuming the pork-based MREs experienced lower feelings of depression (Pork at 24 h: −46.1%; 48 h: −54%; 72 h: −43%; versus Plant at 24 h: −33%) following the ACFT than the plant-based diet, in addition to lower feelings of anger (Pork at 24 h: −31%; 48 h: −42%). Furthermore, when consuming the pork-based diet, the cadets reported better sleep quality by the third day of recovery (24%). Interestingly, a recent meta-analysis by Wirth et al. [51] found no clear relationship between increased protein intake and sleep, suggesting that more studies are needed to better focus on this outcome. However, Zhou and colleagues [52] demonstrated that global sleep scores improved when consuming more protein during an energy deficit among overweight and obese adults. This finding by Zhou et al. [52] may have implications for military and cadet personnel, particularly considering that tactical-specific conditions typically lead to an energy deficit and a catabolic state that necessitate an increased intake of high-quality proteins or even free-form protein EAAs [1]. While our study did not intend to induce an energy deficit state, protein quality may aid recovery (as indicated by perceptions of muscle soreness) and subsequently allow for better perceptions of restful sleep.

### 4.2. Secondary Outcomes

Generally, the argument for the inclusion of plant-based proteins in the diet is centered around a link to reduced chronic disease risk (i.e., cardiovascular disease and cancer) [53]. Li and colleagues [54] found that ingestion of one to two servings of plant-based protein reduced low-density lipoprotein cholesterol, non-high-density lipoprotein, and apolipoprotein B concentrations ≈ 3–4% (for each) in non-hyperlipidemic adults. This corroborates other findings from meta-analyses [55,56] demonstrating that when substituting animal proteins with plant-based proteins, a lipid-lowering effect may be clinically meaningful. While there are many reasons an individual may consider substituting animal proteins with plant-based sources, it does appear that there is a benefit in terms of improving blood lipids [54,56]. We found that following the consumption of the plant-based MRE meals, the cadets experienced reductions in total cholesterol, low-density lipoprotein cholesterol, non-high-density lipoprotein cholesterol, low-density lipoprotein/high-density lipoprotein cholesterol ratio, and total cholesterol/high-density lipoprotein cholesterol ratio over the 3 recovery days. While these changes are transient, it is possible that, in the long term, a diet with more plant-based protein sources may lead to more favorable health outcomes, such as improved blood lipids, than an animal-based protein diet [57]. It is important to note that the other blood clinical panel and safety biomarkers were within normal ranges and did not differ between treatments, suggesting that these two MRE-style regimens did not negatively alter one’s clinical health profile. Regarding the subjective diet satisfaction inventory, we found evidence that when the cadets consumed pork-based MRE meals, they reported a significant increase in appetite and hunger satisfaction. This suggests that the cadets may have had a stronger desire to eat and felt satiated when consuming the pork-based meals regimen. Lastly, our findings demonstrated that neither diet negatively affected self-perceived side effects.

### 4.3. Limitations and Future Directions

The present study is not without limitations. First, this study was limited in assessing the effects of ingesting MRE-style meals before and for 3 days following the performance of the ACFT in college-aged Corps of Cadets involved in military-style training. Second, it was sometimes difficult to coordinate testing sessions and scheduling to take place around the busy schedules of each cadet. Third, the study only included six women (26%), which is similar to the percentage of females in the Corps of Cadets and the U.S. Army (about 19% in 2023). However, this limits the interpretation of sex differences. Future research may benefit from conducting separate studies on men and women to account for sex differences. Additionally, while military assignments do not adjust for menstrual cycle, female participants’ menstrual cycle timing may have affected their ACFT performance. Fourth, our study evaluated the effects of consuming three MREs daily in females and males as per military guidelines. Thus, the relative protein intake differed between sexes (females 2.0 g/kg/d, males 1.6 g/kg/d), which may have influenced results. Fifth, our study only assessed three days of diet intervention and recovery from performing an ACFT. More pronounced benefits may be observed when consuming a diet with higher-quality protein for a longer duration. Finally, due to the need for participants to consume an MRE meal with sufficient time to digest their food, while also considering their class schedules, some testing took place during hotter and less humid times of the day (e.g., 13:00 to 15:00 to 11:00 to 13:00), which may have influenced their performance. Future research should evaluate the effects of consuming higher-quality protein for longer periods of time on military performance and recovery. Additionally, future research should evaluate how differences in the type and amount of protein intake may affect recovery from military-style exercise between sexes. Moreover, since plant-based proteins lack EAAs and creatine compared to animal protein sources, additional research should evaluate how fortifying MREs with EAAs and creatine supplementation may affect military readiness and recovery.

## 5. Conclusions

In comparison to consuming plant-based MREs, consuming pork-based MRE meals can favorably impact perceptions of muscle soreness, reduce urinary markers of protein degradation, attenuate markers of inflammation and stress, promote a more favorable anabolic status (i.e., cortisol/testosterone ratio), and improve perceptions of restful sleep. However, cognitive effects were mixed. Although performance after 3 days of recovery was not affected, these findings provide evidence that individuals who follow a plant-based diet may not recover as well from intense military-style activities as those consuming pork-based protein sources that have higher EAA and creatine content. This is despite consuming recommended amounts of total protein daily for active individuals (i.e., 1.7 g/kg/d). The military should consider using animal-based protein for MREs or fortifying plant-based MREs with EAAs (6–10 g/d) and creatine (2–3 g/d). Given the results observed, it is likely that long-term adherence to plant-based protein diets may result in a significant reduction in performance, fatigue, and/or the need for greater time to recover from intense operations. However, more research is needed to assess how different amounts and types of protein in MREs influence exercise capacity and recovery over time in female and male military personnel.

## Figures and Tables

**Table 1:** Macro- and micronutrient analysis of diets used in the study.

Nutrient	Treatment A (Pork Protein Diet)				Treatment B (Plant Protein Diet)			
	Breakfast	Lunch	Dinner	Total	Breakfast	Lunch	Dinner	Total
Calories	1248	1272	1252	3772	1218	1283	1263	3763
Total Fat [g]	66	55	37	159	59	57	39	155
Saturated Fat [g]	12	18	15	44	12	18	15	45
Trans Fat [g]	0	0	0	0	0	0	0	0
Cholesterol [mg]	81	113	108	302	36	5	0	41
Sodium [mg]	1316	2041	1921	5277	1426	1823	1703	4951
Potassium [mg]	730	1471	1151	3352	600	1450	1130	3180
Total Carbohydrates [g]	137	153	200	490	142	161	108	510
Dietary Fiber [g]	6	14	32	51	7	16	34	56
Sugars [g]	43	32	58	132	44	31	57	132
Protein [g]	26	49	51	126	32	47	49	128
Histidine †	0.4	0.5	0.7	1.5	0.2	0.3	0.5	0.9
Isoleucine †*	0.5	0.7	1.1	2.3	0.2	0.4	0.8	1.4
Leucine †*	**1.0**	**1.3**	**2.0**	**4.3**	**0.5**	**0.7**	**1.5**	**2.6**
Lysine †	0.7	0.8	1.6	3.1	0.2	0.4	1.2	1.8
Methionine † + Cystine	0.4	0.5	0.7	1.7	0.2	0.3	0.5	1.0
Phenylalanine † + Tyrosine	1.1	1.5	2.1	4.7	0.6	1.9	1.5	4.0
Threonine †	0.4	0.6	1.0	2.0	0.2	0.4	0.8	1.3
Tryptophan †	0.1	0.2	0.3	0.6	0.1	0.1	0.2	0.4
Valine †*	0.6	0.9	1.3	2.8	0.2	0.5	1.0	1.7
Protein Quality Score	55%	32%	57%	48%	12%	16%	44%	27%
Essential Amino Acids (g)	5.2	6.9	10.8	22.9	2.2	5.0	8.0	15.2
Protein (g/kg)	0.34	0.66	0.68	1.69	0.42	0.63	0.66	1.71
EAA (g/kg)	0.07	0.09	0.14	0.31	0.03	0.07	0.11	0.20
Creatine (g)	0.226	0.831	0.76	1.817	0.126	0.08	0.009	0.215
Creatine (g/kg)	0.003	0.011	0.010	0.024	0.002	0.001	0.000	0.003

† Essential Amino Acids (EAAs); * denotes the “branched chain amino acids”.

## Data Availability

Data and statistical analyses are available for non-commercial scientific inquiry and/or educational use upon request to the corresponding author if the use of data does not violate IRB restrictions and sponsored research agreements and the authors and sponsors of this work are appropriately acknowledged.

## Supplementary Materials

The following supporting information can be downloaded at: https://www.mdpi.com/article/10.3390/nu17121995/s1. Table S1: Demographic data; Table S2: Anthropometric and resting hemodynamic data; Table S3: Performance-related data; Table S4: Army Combat Fitness Test performance scores data; Table S5: Muscle soreness rating results; Table S6: Trail Making Task Test data; Table S7: Psychomotor Vigilance Task Test changes; Table S8: Readiness to Perform questionnaire results; Table S9: Profile of Mood States results; and Table S10: While blood cell blood count results; Table S11: Serum renal function and electrolyte results; Table S12: Serum blood lipid results; Table S13: Markers of protein degradation and muscle and liver enzymes; Table S14: Hormonal markers of catabolism and anabolism; Table S15: Inflammatory marker results; Table S16: Urinary markers of catabolism; Table S17: Diet satisfaction inventory; and Table S18: Frequency and severity of side effect. Figure S1: Changes in Readiness to Perform questionnaire results; Figure S2: Percent changes in Profile of Mood States categories; Figure S3a: Percent changes in white and red cells; Figure S3b: Percent changes in whole blood white blood cells; Figure S4: Percent changes renal function and electrolyte-related variables; Figure S5: Percent changes in markers of catabolism and enzymes; Figure S6: Percent changes in cytokines; and Figure S7: Percent changes in cytokines. Data are displayed as mean percentage changes from baseline with 95% confidence intervals.
