# Supplementary material for: Effects of Pork Protein Ingestion Prior to and Following Performing the Army Combat Fitness Test on Markers of Catabolism, Inflammation, and Recovery
Source: Nutrients. 2025 Jun 13;17(12):1995. doi: 10.3390/nu17121995 (PMC12195741; doi:10.3390/nu17121995)
Supplement: Supplementary file 1 [file nutrients-17-01995-s001.zip › Supplemental Tables and Figures R1.pdf]

Table S1. Demographic Data

Variable	Treatment	Sex	*n*	Mean		p-Value
Age	Pork	Male	*17*	20.2 ± 2.4	Treatment	1.000
(yrs)		Female	*6*	19.5 ± 1.2	Sex	0.351
		Total	*23*	20.0 ± 2.1		
	Plant	Male	*17*	20.2 ± 2.4		
		Female	*6*	19.5 ± 1.2		
		Total	*23*	20.0 ± 2.1		
	Total	Male	*34*	20.2 ± 2.3		
		Female	*12*	19.5 ± 1.2		
		Total	*46*	20.0 ± 2.1		
Height	Pork	Male	*17*	177.1 ± 5.8	Treatment	0.998
(cm)		Female	*6*	161.8 ± 3.5	Sex	<.001
		Total	*23*	173.1 ± 8.6		
	Plant	Male	*17*	177.1 ± 5.8		
		Female	*6*	161.8 ± 3.5		
		Total	*23*	173.1 ± 8.6		
	Total	Male	*34*	177.1 ± 5.7		
		Female	*12*	161.8 ± 3.3		
		Total	*46*	173.1 ± 8.5		
Weight	Pork	Male	*17*	79.0 ± 9.4	Treatment	0.867
(kg)		Female	*6*	62.6 ± 4.8	Sex	<.001
		Total	*23*	74.7 ± 11.1		
	Plant	Male	*17*	79.0 ± 9.1		
		Female	*6*	63.6 ± 5.1		
		Total	*23*	75.0 ± 10.7		
	Total	Male	*34*	79.0 ± 9.1		
		Female	*12*	63.1 ± 4.8		
		Total	*46*	74.9 ± 10.8		
Body Mass Index	Pork	Male	*17*	25.2 ± 3.0	Treatment	0.839
(kg/m^2)		Female	*6*	23.9 ± 1.3	Sex	0.219
		Total	*23*	24.9 ± 2.7		
	Plant	Male	*17*	25.2 ± 2.9		
		Female	*6*	24.3 ± 1.5		
		Total	*23*	25.0 ± 2.6		
	Total	Male	*34*	25.2 ± 2.9		
		Female	*12*	24.1 ± 1.3		
		Total	*46*	24.9 ± 2.6		
Resting Heart Rate	Pork	Male	*17*	62.9 ± 12.3	Treatment	0.922
(bpm)		Female	*6*	66.3 ± 10.3	Sex	0.125
		Total	*23*	63.8 ± 11.7		
	Plant	Male	*17*	60.6 ± 10.7		
		Female	*6*	69.3 ± 12.9		
		Total	*23*	62.9 ± 11.7		
	Total	Male	*34*	61.8 ± 11.4		
		Female	*12*	67.8 ± 11.3		
		Total	*46*	63.3 ± 11.5		
Systolic Blood Pressure	Pork	Male	*17*	116.9 ± 9.3	Treatment	0.415
(mmHg)		Female	*6*	102.7 ± 3.9	Sex	<.001
		Total	*23*	113.2 ± 10.4		
	Plant	Male	*17*	117.8 ± 7.5		
		Female	*6*	106.0 ± 4.3		
		Total	*23*	114.7 ± 8.5		
	Total	Male	*34*	117.3 ± 8.3		
		Female	*12*	104.3 ± 4.3		
		Total	*46*	113.9 ± 9.4		
Diastolic Blood Pressure	Pork	Male	*17*	68.9 ± 7.2	Treatment	0.281
(mmHg)		Female	*6*	64.0 ± 6.2	Sex	0.089
		Total	*23*	67.7 ± 7.2		
	Plant	Male	*17*	70.3 ± 6.7		
		Female	*6*	67.5 ± 4.4		
		Total	*23*	69.6 ± 6.2		
	Total	Male	*34*	69.6 ± 6.9		
		Female	*12*	65.8 ± 5.4		
		Total	*46*	68.6 ± 6.7		

Data are expressed as means ± standard deviations for the pork and soy protein treatments.

Table S2. Anthropometric and resting hemodynamic changes.

Variable	Treatmen	*n*	Time Point<br>Baseline	24-hour	48-hour	72-hour	Mean<br>(SEM)	Effect	p-Value	η_p^2
Weight	Pork	*23*	74.7 ± 11.1	74.8 ± 11.0	75.1 ± 11.1 †	75.0 ± 11.0	74.9 ± 2.2	T	0.008	0.101
(kg)	Plant	*23*	75.0 ± 10.7	74.8 ± 10.3	75.2 ± 10.5	75.0 ± 10.5	75.0 ± 2.2	T x T	0.571	0.013
	Total	*46*	74.9 ± 10.8	74.8 ± 10.6	75.2 ± 10.7 †	75.0 ± 10.7	74.9 ± 1.6			
Body Mass Index	Pork	*23*	24.9 ± 2.7	24.9 ± 2.7	25.0 ± 2.8 †	25.0 ± 2.8	24.9 ± 0.6	T	0.007	0.105
(kg/m^2)	Plant	*23*	25.0 ± 2.6	24.9 ± 2.5	25.1 ± 2.6	25.0 ± 2.6	25.0 ± 0.6	T x T	0.564	0.013
	Total	*46*	24.9 ± 2.6	24.9 ± 2.6	25.0 ± 2.6 †	25.0 ± 2.7	25.0 ± 0.4			
Resting Heart Rate	Pork	*23*	63.8 ± 11.7	61.5 ± 9.9	62.6 ± 8.4	65.8 ± 11.5	114.3 ± 1.7	T	0.575	0.014
(bpm)	Plant	*23*	62.9 ± 11.7	65.7 ± 10.4	62.7 ± 9.7	63.3 ± 8.7	114.6 ± 1.7	T x T	0.110	0.045
	Total	*46*	63.3 ± 11.5	63.6 ± 10.2	62.7 ± 8.9	64.6 ± 10.1	114.5 ± 1.2			
Systolic Blood Pressure	Pork	*23*	113.2 ± 10.4	114.0 ± 8.7	114.8 ± 10.6	115.3 ± 8.5	68.4 ± 0.9	T	0.737	0.009
(mmHg)	Plant	*23*	114.7 ± 8.5	114.1 ± 11.7	114.3 ± 10.3	115.2 ± 9.0	68.9 ± 0.9	T x T	0.875	0.005
	Total	*46*	113.9 ± 9.4	114.0 ± 10.2	114.6 ± 10.4	115.3 ± 8.7	68.7 ± 0.6			
Diastolic Blood Pressure	Pork	*23*	67.7 ± 7.2	68.6 ± 5.7	68.4 ± 6.5	69.1 ± 5.6	63.4 ± 1.8	T	0.937	0.003
(mmHg)	Plant	*23*	69.6 ± 6.2	68.7 ± 5.5	68.5 ± 4.4	69.0 ± 5.7	63.7 ± 1.8	T x T	0.725	0.010
	Total	*46*	68.6 ± 6.7	68.7 ± 5.6	68.5 ± 5.5	69.1 ± 5.6	63.5 ± 1.3			

Data are expressed as means ± standard deviations for the the pork and Plant protein treatments. Data were analyzed using a multivariate and univariate General Linear Model with repeated measures. P-levels, with partial ETA squared (η_p^2) effect size, were reported. General Linear Model analysis revealed no significant overall Wilk's Lambda for Time ($p = 0.140$, $\eta_p^2 = 0.052$) and no significant Treatment x Time ($p = 0.830$, $\eta_p^2 = 0.025$) effect. Greenhouse-Geisser univariate p-levels are listed for time (T) and treatment x time (T x T) interaction effects. Significance was determined via pairwise comparison, with LSD posthoc adjustment, indicated as differences from baseline: † = $p < 0.05$ [‡ = $p > 0.05$ to $p < 0.10$]. η_p^2 effect size values of 0.01 - 0.05 = small, 0.06 - 0.13 = medium, and >0.14 = large.

Table S3. Performance related data.

Variable	Treatment	*n*	Baseline	72-hour		Mean (SEM)		Effect	p-Value	η_p^2
Vertical Jump (in)	Pork	*23*	20.5 ± 4.7	20.3 ± 4.8		20.4 ± 1.0		T	0.510	0.010
	Plant	*23*	20.3 ± 4.4	20.2 ± 4.9		20.3 ± 1.0		T x T	0.719	0.003
	Total	*46*	20.4 ± 4.5	20.2 ± 4.8		20.3 ± 0.7				
Peak Power (watts)	Pork	*23*	7739 ± 999	7707 ± 965		7723 ± 203		T	0.584	0.007
	Plant	*23*	7714 ± 946	7703 ± 1015		7708 ± 203		T x T	0.786	0.002
	Total	*46*	7726 ± 962	7705 ± 979		7716 ± 144				
3-Repetition Maximum Deadlift (lbs)	Pork	*23*	246.9 ± 78.5	250.7 ± 73.3		248.8 ± 15.5		T	0.063	0.076
	Plant	*23*	248.6 ± 71.6	255.0 ± 75.4	‡	251.8 ± 15.5		T x T	0.625	0.005
	Total	*46*	247.7 ± 74.3	252.8 ± 73.5	‡	250.3 ± 10.9	**			
Standing Power Throw (m)	Pork	*23*	8.6 ± 2.4	8.3 ± 2.6		8.5 ± 0.5		T	0.624	0.005
	Plant	*23*	8.6 ± 2.5	8.7 ± 2.6		8.6 ± 0.5		T x T	0.257	0.029
	Total	*46*	8.6 ± 2.4	8.5 ± 2.6		8.6 ± 0.4				
Hand Release Push-up (total repetitions)	Pork	*23*	48.8 ± 8.6	50.9 ± 7.9	†	49.8 ± 1.7		T	<.001	0.244
	Plant	*23*	47.1 ± 6.9	49.8 ± 9.4	†	48.4 ± 1.7		T x T	0.661	0.004
	Total	*46*	47.9 ± 7.8	50.3 ± 8.6	†	49.1 ± 1.2	*			
Sprint-Drag-Carry (sec)	Pork	*23*	107.0 ± 31.1	109.7 ± 20.1		108.3 ± 4.5		T	0.659	0.004
	Plant	*23*	108.3 ± 18.9	107.5 ± 18.0		107.9 ± 4.5		T x T	0.419	0.015
	Total	*46*	107.6 ± 25.4	108.6 ± 18.9		108.1 ± 3.2				
Plank Test (sec)	Pork	*23*	152.1 ± 51.4	171.5 ± 43.3	†	161.8 ± 9.6		T	0.003	0.188
	Plant	*23*	150.3 ± 48.7	166.7 ± 54.6	†	158.5 ± 9.6		T x T	0.793	0.002
	Total	*46*	151.2 ± 49.5	169.1 ± 48.8	†	160.1 ± 6.8	*			
Two-Mile Run (minutes)	Pork	*23*	17.3 ± 2.4	16.9 ± 2.0	‡	17.1 ± 0.4		T	0.001	0.214
	Plant	*23*	17.2 ± 2.3	16.5 ± 2.1	†	16.8 ± 0.4		T x T	0.400	0.016
	Total	*46*	17.2 ± 2.4	16.7 ± 2.0	†	17.0 ± 0.3	*			
Total Score (Percentile)	Pork	*23*	17.3 ± 2.4	16.9 ± 2.0	‡	17.1 ± 0.4		T	0.001	0.214
	Plant	*23*	17.2 ± 2.3	16.5 ± 2.1	†	16.8 ± 0.4		T x T	0.400	0.016
	Total	*46*	17.2 ± 2.4	16.7 ± 2.0	†	17.0 ± 0.3	*			

Data are expressed as means ± standard deviations for the the pork and Plant protein treatments. Data were analyzed using a multivariate and univariate General Linear Model with repeated measures. P-levels, with partial ETA squared (η_p^2) effect size, were reported. General Linear Model analysis revealed a significant overall Wilk's Lambda for Time ($p < 0.004$, η_p^2=0.464) and no significant Treatment x Time (p = 0.378, η_p^2=0.218) effect. Greenhouse-Geisser univariate p-levels are listed for time (T) and treatment x time (T x T) interaction effects. Significance was determined via pairwise comparison, with LSD posthoc adjustment, indicated as differences from baseline: † = p < 0.05 [‡ = p > 0.05 to p < 0.10]. η_p^2 effect size values of 0.01 - 0.05 = small, 0.06 - 0.13 = medium, and >0.14 = large.

Table S4. Army Combat Fitness Test Performance Scores data.

Variable	Treatmer	*n*	Baseline	72-hour	Mean (SEM)	Effect	p-Value	η_p^2
3-Repetition Maximum Deadlift	Pork	*23*	85.0 ± 13.3	96.3 ± 47.7	90.6 ± 4.1	T	0.232	0.032
(points)	Plant	*23*	87.2 ± 10.5	88.0 ± 10.6	87.6 ± 4.1	T x T	0.301	0.024
	Total	*46*	86.1 ± 11.9	92.1 ± 34.4	89.1 ± 2.9			
Standing Power Throw	Pork	*23*	76.7 ± 18.1	77.3 ± 17.8	77.0 ± 3.3	T	0.461	0.012
(points)	Plant	*23*	80.0 ± 14.3	80.9 ± 13.5	80.4 ± 3.3	T x T	0.849	0.001
	Total	*46*	78.3 ± 16.2	79.1 ± 15.7	78.7 ± 2.3			
Hand Release Push-up	Pork	*23*	93.7 ± 6.4	95.4 ± 5.5 †	94.6 ± 1.3	T	0.006	0.161
(points)	Plant	*23*	93.3 ± 6.1	94.2 ± 6.8	93.8 ± 1.3	T x T	0.389	0.017
	Total	*46*	93.5 ± 6.2	94.8 ± 6.1 †	94.2 ± 0.9			
Sprint-Drag-Carry	Pork	*23*	90.4 ± 9.2	91.4 ± 8.1	90.9 ± 1.5	T	0.309	0.024
(points)	Plant	*23*	92.2 ± 6.7	92.9 ± 6.1	92.5 ± 1.5	T x T	0.829	0.001
	Total	*46*	91.3 ± 8.0	92.1 ± 7.1	91.7 ± 1.1			
Plank Test	Pork	*23*	78.9 ± 17.4	84.8 ± 13.4 ‡	81.9 ± 3.4	T	0.016	0.125
(points)	Plant	*23*	75.9 ± 19.7	81.6 ± 20.8 ‡	78.7 ± 3.4	T x T	0.970	0.000
	Total	*46*	77.4 ± 18.4	83.2 ± 17.4 †	80.3 ± 2.4			
Two-Mile Run	Pork	*23*	82.2 ± 12.0	84.7 ± 10.2 ‡	83.4 ± 2.3	T	0.003	0.186
(points)	Plant	*23*	83.6 ± 13.0	87.3 ± 10.3 †	85.5 ± 2.3	T x T	0.496	0.011
	Total	*46*	82.9 ± 12.4	86.0 ± 10.2 †	84.4 ± 1.6			
Total Score	Pork	*23*	507.0 ± 46.5	529.9 ± 60.0 †	518.4 ± 9.0	T	0.004	0.170
(points)	Plant	*23*	512.1 ± 41.6	525.0 ± 39.4	518.5 ± 9.0	T x T	0.405	0.016
	Total	*46*	509.5 ± 43.7	527.4 ± 50.3 †	518.5 ± 6.3			

Data are expressed as means ± standard deviations for the the pork and Plant protein treatments. Data were analyzed using a multivariate and univariate General Linear Model with repeated measures. P-levels, with partial ETA squared (η_p^2) effect size, were reported. General Linear Model analysis revealed a significant overall Wilk's Lambda for Time (p < 0.009, η_p^2=0.341) and no significant Treatment x Time (p = 0.866, η_p^2=0.060) effect. Greenhouse-Geisser univariate p-levels are listed for time (T) and treatment x time (T x T) interaction effects. Significance was determined via pairwise comparison, with LSD posthoc adjustment, indicated as differences from baseline: † = p < 0.05 [‡ = p > 0.05 to p < 0.10].

Table S5. Muscle soreness rating results.

Variable	Treatment	*n*	Baseline	24-hour	48-hour	72-hour	Mean (SEM)	Effect	p-Value	η_p^2
				Time Point						
Lower Medial Thigh (mm)	Pork	*23*	34.3 ± 29.7	38.8 ± 33.5	45.7 ± 34.9	41.3 ± 32.9	40.0 ± 5.2	T	0.007	0.090
	Plant	*23*	26.8 ± 26.6	41.8 ± 33.3 †	46.1 ± 32.1 †	32.6 ± 22.1	36.8 ± 5.2	T x T	0.444	0.020
	Total	*46*	30.5 ± 28.2	40.3 ± 33.1 †	45.9 ± 33.1 †	36.9 ± 28.0	38.4 ± 3.7			
Lower Lateral Thigh (mm)	Pork	*23*	28.1 ± 27.2	22.3 ± 18.4	34.7 ± 32.9	29.3 ± 25.1	28.6 ± 3.7	T	0.040	0.065
	Plant	*23*	14.4 ± 13.7	22.8 ± 20.4 ‡	27.1 ± 25.6 †	16.3 ± 15.1	20.2 ± 3.7	T x T	0.177	0.038
	Total	*46*	21.2 ± 22.4 *	22.6 ± 19.2	30.9 ± 29.4 †	22.8 ± 21.5 *	24.4 ± 2.6			
Mid Thigh (mm)	Pork	*23*	11.0 ± 14.9	12.6 ± 15.3	11.7 ± 12.0	13.5 ± 17.9	12.2 ± 2.0	T	0.206	0.034
	Plant	*23*	3.8 ± 4.9	8.8 ± 10.2	11.0 ± 13.5 †	8.5 ± 8.7	8.0 ± 2.0	T x T	0.437	0.020
	Total	*46*	7.4 ± 11.6 *	10.7 ± 13.0	11.3 ± 12.6 ‡	11.0 ± 14.1	10.1 ± 1.4			

Data are expressed as means ± standard deviations for the the pork and Plant protein treatments. Data were analyzed using a multivariate and univariate General Linear Model with repeated measures. P-levels, with partial ETA squared (η_p^2) effect size, were reported. General Linear Model analysis revealed a significant overall Wilk's Lambda for Time ($p = 0.027$, $\eta_p^2 = 0.047$) and no significant Treatment x Time ($p = 0.553$, $\eta_p^2 = 0.020$) effect. Greenhouse-Geisser univariate p-levels are listed for time (T) and treatment x time (T x T) interaction effects. Significance was determined via pairwise comparison, with LSD posthoc adjustment, indicated as differences from baseline: † = $p < 0.05$ [‡ = $p > 0.05$ to $p < 0.10$]. η_p^2 effect size values of 0.01 - 0.05 = small, 0.06 - 0.13 = medium, and >0.14 = large.

Table S6. Trail making task changes.

Variable	Treatment	*n*	Baseline	24-hour	48-hour	72-hour	Mean (SEM)	Effect	p-Value	η_p^2
				Time Point						
Task A (sec)	Pork	*23*	15.0 ± 3.2	13.1 ± 2.9 †	12.8 ± 3.2 †	11.7 ± 2.5 †	13.2 ± 0.6	T	0.000	0.386
	Plant	*23*	14.5 ± 3.5	13.5 ± 3.0 ‡	12.9 ± 4.0 †	12.1 ± 2.8 †	13.2 ± 0.6	T x T	0.455	0.019
	Total	*46*	14.8 ± 3.3	13.3 ± 2.9 †	12.8 ± 3.6 †	11.9 ± 2.6 †	13.2 ± 0.4			
Task B (sec)	Pork	*23*	29.7 ± 9.6	26.3 ± 13.3	26.0 ± 18.3	21.0 ± 9.4 †	25.7 ± 2.2	T	0.000	0.214
	Plant	*23*	34.3 ± 14.2	26.3 ± 10.6 †	23.2 ± 12.8 †	22.4 ± 13.4 †	26.5 ± 2.2	T x T	0.215	0.034
	Total	*46*	32.0 ± 12.2	26.3 ± 11.9 †	24.6 ± 15.7 †	21.7 ± 11.5 †	26.1 ± 1.6			

Data are expressed as means ± standard deviations for the the pork and Plant protein treatments. Data were analyzed using a multivariate and univariate General Linear Model with repeated measures. P-levels, with partial ETA squared (η_p^2) effect size, were reported. General Linear Model analysis revealed a significant overall Wilk's Lambda for Time ($p < 0.001$, $\eta_p^2 = 0.281$) and no significant Treatment x Time ($p = 0.330$, $\eta_p^2 = 0.026$) effect. Greenhouse-Geisser univariate p-levels are listed for time (T) and treatment x time (T x T) interaction effects. Significance was determined via pairwise comparison, with LSD posthoc adjustment, indicated as differences from baseline: † = $p < 0.05$ [‡ = $p > 0.05$ to $p < 0.10$]. η_p^2 effect size values of 0.01 - 0.05 = small, 0.06 - 0.13 = medium, and >0.14 = large.

Table S7. Psychomotor vigilance task changes.

Variable	Treatment	*n*	Baseline	24-hour	48-hour	72-hour	Mean (SEM)	Effect	p-Value	η_p^2
					Time Point					
Targets Correct (%)	Pork	*23*	90.5 ± 7.1	89.1 ± 8.0	87.2 ± 12.5	87.3 ± 8.7 †	88.5 ± 1.8	T	0.339	0.025
	Plant	*23*	87.6 ± 8.0	87.5 ± 10.2	86.4 ± 13.9	88.4 ± 9.1	87.5 ± 1.8	G x T	0.454	0.018
	Total	*46*	89.1 ± 7.6	88.3 ± 9.1	86.8 ± 13.1	87.9 ± 8.8	88.0 ± 1.2			
Correct Reaction Time (ms)	Pork	*23*	464 ± 30	474 ± 30 ‡	481 ± 32 †	477 ± 32 †	474 ± 6	T	0.195	0.035
	Plant	*23*	495 ± 29	492 ± 33	491 ± 32	483 ± 30 †	490 ± 6	G x T	0.002	0.111
	Total	*46*	479 ± 33 *	483 ± 32 †*	486 ± 32 †	480 ± 31 *	482 ± 4 ⁑			
False Alarms (%)	Pork	*23*	1.9 ± 1.6	2.5 ± 2.2	2.9 ± 2.6 ‡	3.1 ± 2.2 †	2.6 ± 0.4	T	0.193	0.036
	Plant	*23*	3.1 ± 2.5	3.1 ± 2.3 ‡	3.7 ± 3.4	2.8 ± 2.6	3.2 ± 0.4	G x T	0.221	0.033
	Total	*46*	2.5 ± 2.2 ⁑	2.8 ± 2.2 †	3.3 ± 3.0 †	3.0 ± 2.4	2.9 ± 0.3			

Data are expressed as means ± standard deviations for the the pork and Plant protein treatments. Data were analyzed using a multivariate and univariate General Linear Model with repeated measures. P-levels, with partial ETA squared (η_p^2) effect size, were reported. General Linear Model analysis revealed no significant overall Wilk's Lambda for Time ($p = 0.472$, $\eta_p^2 = 0.022$) and a significant Treatment x Time ($p = 0.038$, $\eta_p^2 = 0.044$) effect. Greenhouse-Geisser univariate p-levels are listed for time (T) and treatment x time (G x T) interaction effects. Significance was determined via pairwise comparison, with LSD posthoc adjustment, indicated as differences from baseline: † = $p < 0.05$ [‡ = $p > 0.05$ to $p < 0.10$]. η_p^2 effect size values of 0.01 - 0.05 = small, 0.06 - 0.13 = medium, and >0.14 = large.

Table S8. Readiness to Perform questionnaire.

					Time Point		Mean			
**Variable**	**Treatment**	***n***	**Baseline**	**24-hour**	**48-hour**	**72-hour**	**(SEM)**	**Effect**	**p-Value**	**η_p^2**
I slept well last night.	Pork	*23*	3.22 ± 0.80	3.41 ± 0.96	3.22 ± 0.99	3.54 ± 1.01	3.35 ± 0.15	T	0.098	0.049
	Plant	*23*	3.35 ± 0.83	3.44 ± 0.83	2.88 ± 1.06 †	3.08 ± 1.06	3.19 ± 0.15	G x T	0.155	0.040
	Total	*46*	3.28 ± 0.81	3.42 ± 0.89	3.05 ± 1.03	3.31 ± 1.05	3.27 ± 0.11			
I am looking forward to today's workout.	Pork	*23*	3.64 ± 0.84	3.20 ± 0.81 †	3.26 ± 0.77 ‡	3.57 ± 0.68	3.42 ± 0.11	T	0.044	0.068
	Plant	*23*	3.48 ± 0.67	3.28 ± 0.57	3.23 ± 0.54	3.17 ± 0.84 ‡	3.29 ± 0.11	G x T	0.224	0.033
	Total	*46*	3.56 ± 0.76	3.24 ± 0.69 †	3.25 ± 0.66 †	3.37 ± 0.78 ⁑	3.35 ± 0.08			
I am optimistic about my future performance.	Pork	*23*	4.13 ± 0.69	3.78 ± 0.65 †	3.86 ± 0.65 ‡	3.75 ± 0.78 †	3.88 ± 0.12	T	0.007	0.096
	Plant	*23*	3.91 ± 0.67	3.80 ± 0.75	3.68 ± 0.74	3.59 ± 0.81 ‡	3.75 ± 0.12	G x T	0.613	0.012
	Total	*46*	4.02 ± 0.68	3.79 ± 0.70 †	3.77 ± 0.70 †	3.67 ± 0.79 †	3.81 ± 0.09			
I feel vigorous and energetic.	Pork	*23*	3.24 ± 0.67	3.19 ± 0.81	3.20 ± 0.91	3.15 ± 0.95	3.20 ± 0.13	T	0.839	0.006
	Plant	*23*	3.00 ± 0.67	3.03 ± 0.86	2.95 ± 0.93	2.87 ± 0.88	2.96 ± 0.13	G x T	0.974	0.001
	Total	*46*	3.12 ± 0.68	3.11 ± 0.83	3.08 ± 0.92	3.01 ± 0.91	3.08 ± 0.10			
My appetite is great.	Pork	*23*	3.83 ± 0.65	3.81 ± 0.74	3.96 ± 0.72	3.93 ± 0.80	3.88 ± 0.14	T	0.220	0.033
	Plant	*23*	4.09 ± 0.73	3.65 ± 0.97 †	3.53 ± 1.06 †	3.75 ± 1.00 ‡	3.75 ± 0.14	G x T	0.047	0.061
	Total	*46*	3.96 ± 0.70	3.73 ± 0.86 ‡	3.74 ± 0.92	3.84 ± 0.90	3.82 ± 0.10			
I have little muscle soreness.	Pork	*23*	3.57 ± 1.12	3.28 ± 0.94	3.37 ± 0.96	3.43 ± 0.93	3.41 ± 0.12	T	0.194	0.036
	Plant	*23*	3.57 ± 1.20	3.26 ± 0.83	2.95 ± 0.92 ‡	3.38 ± 0.95	3.29 ± 0.12	G x T	0.636	0.012
	Total	*46*	3.57 ± 1.15	3.27 ± 0.88	3.16 ± 0.95 ‡	3.41 ± 0.93	3.35 ± 0.09			

Data are expressed as means ± standard deviations for the the pork and Plant protein treatments. Data were analyzed using a multivariate and univariate General Linear Model with repeated measures. P-levels, with partial ETA squared (η_p^2) effect size, were reported. General Linear Model analysis revealed a significant overall Wilk's Lambda for Time (p = 0.002, η_p^2 = 0.097) and a non-significant Treatment x Time (p = 0.148, η_p^2 = 0.060) effect. Greenhouse-Geisser univariate p-levels are listed for time (T) and treatment x time (G x T) interaction effects. Significance was determined via pairwise comparison, with LSD posthoc adjustment, indicated as differences from baseline: † = $p < 0.05$ [‡ = $p > 0.05$ to $p < 0.10$]. η_p^2 effect size values of 0.01 - 0.05 = small, 0.06 - 0.13 = medium, and >0.14 = large.

Table S9. Profile of Mood States results.

					Time Point		Mean			
**Variable**	**Treatment**	***n***	**Baseline**	**24-hour**	**48-hour**	**72-hour**	**(SEM)**	**Effect**	**p-Value**	**η_p^2**
Tension	Pork	*23*	6.22 ± 4.57	5.65 ± 4.60	4.65 ± 4.67 †	4.91 ± 4.60 ‡	5.36 ± 0.94	T	0.003	0.102
	Plant	*23*	6.52 ± 5.78	5.17 ± 4.10 ‡	5.17 ± 5.50	4.70 ± 4.85 †	5.39 ± 0.94	G x T	0.654	0.012
	Total	*46*	6.37 ± 5.16	5.41 ± 4.31 ‡	4.91 ± 5.05 †	4.80 ± 4.67 †	5.38 ± 0.66			
Depression	Pork	*23*	5.30 ± 6.71	4.04 ± 7.06 †	3.57 ± 6.71 ‡	3.52 ± 6.27 ‡	4.11 ± 1.29	T	0.053	0.066
	Plant	*23*	5.00 ± 6.42	3.87 ± 6.06 ‡	4.57 ± 6.91	4.00 ± 5.92	4.36 ± 1.29	G x T	0.474	0.016
	Total	*46*	5.15 ± 6.50	3.96 ± 6.50 †	4.07 ± 6.75	3.76 ± 6.03 †	4.23 ± 0.91			
Anger	Pork	*23*	4.61 ± 6.04	4.04 ± 5.76	3.26 ± 4.62 ‡	4.04 ± 6.01	3.99 ± 0.94	T	0.152	0.040
	Plant	*23*	4.30 ± 4.59	2.61 ± 3.14	3.61 ± 5.72 †	2.87 ± 3.68	3.35 ± 0.94	G x T	0.353	0.024
	Total	*46*	4.46 ± 5.30	3.33 ± 4.64 †	3.43 ± 5.15 ‡	3.46 ± 4.97	3.67 ± 0.66			
Fatigue	Pork	*23*	6.22 ± 3.94	6.35 ± 4.46	6.30 ± 4.53	5.35 ± 5.62	6.05 ± 0.79	T	0.593	0.014
	Plant	*23*	6.39 ± 4.67	5.87 ± 3.42	6.52 ± 4.79	6.00 ± 4.38	6.20 ± 0.79	G x T	0.796	0.007
	Total	*46*	6.30 ± 4.27	6.11 ± 3.93	6.41 ± 4.61	5.67 ± 4.99	6.13 ± 0.56			
Confusion	Pork	*23*	5.96 ± 4.44	5.74 ± 4.74	4.96 ± 4.75	5.43 ± 5.30	5.52 ± 0.91	T	0.063	0.057
	Plant	*23*	5.83 ± 4.30	4.39 ± 4.03 †	4.87 ± 5.01	4.52 ± 4.68 ‡	4.90 ± 0.91	G x T	0.308	0.027
	Total	*46*	5.89 ± 4.32	5.07 ± 4.40 †	4.91 ± 4.83 †	4.98 ± 4.96 ‡	5.21 ± 0.65			
Vigor	Pork	*23*	13.87 ± 5.78	11.43 ± 6.98 †	11.17 ± 8.77 †	10.87 ± 8.10 †	11.84 ± 1.46	T	<.001	0.225
	Plant	*23*	14.04 ± 6.53	10.65 ± 8.12 †	9.96 ± 8.00 †	9.83 ± 7.51 †	11.12 ± 1.46	G x T	0.705	0.010
	Total	*46*	13.96 ± 6.10	11.04 ± 7.50 †	10.57 ± 8.33 †	10.35 ± 7.74 †	11.48 ± 1.03			
Total Mood Disturbance Score	Pork	*23*	42.2 ± 24.7	37.3 ± 26.6 †	33.9 ± 24.7 †	34.1 ± 25.7 †	36.9 ± 5.0	T	<.001	0.196
	Plant	*23*	42.1 ± 25.2	32.6 ± 21.2 †	34.7 ± 27.5 †	31.9 ± 24.4 †	35.3 ± 5.0	G x T	0.404	0.021
	Total	*46*	42.1 ± 24.7	34.9 ± 23.9 †	34.3 ± 25.9 †	33.0 ± 24.8 †	36.1 ± 3.5			

Data are expressed as means ± standard deviations for the the pork and Plant protein treatments. Data were analyzed using a multivariate and univariate General Linear Model with repeated measures. P-levels, with partial ETA squared (η_p^2) effect size, were reported. General Linear Model analysis revealed a significant overall Wilk's Lambda for Time (p < 0.001, η_p^2 = 0.128) and a non-significant Treatment x Time (p = 0.927, η_p^2 = 026) effect. Greenhouse-Geisser univariate p-levels are listed for time (T) and treatment x time (G x T) interaction effects. Significance was determined via pairwise comparison, with LSD posthoc adjustment, indicated as differences from baseline: † = $p < 0.05$ [‡ = $p > 0.05$ to $p < 0.10$]. η_p^2 effect size values of 0.01 - 0.05 = small, 0.06 - 0.13 = medium, and >0.14 = large.

Table S10. Whole blood cell blood count results.

					Time Point		Mean			
**Variable**	**Treatmer**	***n***	**Baseline**	**24-hour**	**48-hour**	**72-hour**	**(SEM)**	**Effect**	**p-Value**	**η_p^2**
White Blood Cells	Pork	*23*	6.70 ± 2.18	6.74 ± 1.63	7.17 ± 1.93	6.95 ± 1.90	6.89 ± 0.27	T	0.770	0.007
(K/µL)	Plant	*23*	6.49 ± 1.01	6.67 ± 1.19	6.37 ± 1.39	6.64 ± 1.42	6.54 ± 0.27	T x T	0.404	0.021
	Total	*46*	6.59 ± 1.68	6.71 ± 1.41	6.77 ± 1.71	6.80 ± 1.66	6.72 ± 0.19			
Red Blood Cells	Pork	*23*	4.70 ± 0.40	4.69 ± 0.42	4.71 ± 0.48	4.76 ± 0.48	4.72 ± 0.09	T	0.525	0.015
(M/µL)	Plant	*23*	4.74 ± 0.41	4.70 ± 0.45	4.67 ± 0.47	4.69 ± 0.49	4.70 ± 0.09	T x T	0.245	0.031
	Total	*46*	4.72 ± 0.40	4.69 ± 0.43	4.69 ± 0.47	4.72 ± 0.48	4.71 ± 0.06			
Hemoglobin	Pork	*23*	14.26 ± 1.19	14.20 ± 1.20	14.22 ± 1.25	14.37 ± 1.40	14.26 ± 0.25	T	0.302	0.027
(g/dL)	Plant	*23*	14.32 ± 1.20	14.10 ± 1.22	‡ 14.16 ± 1.28	14.15 ± 1.36	14.18 ± 0.25	T x T	0.384	0.022
	Total	*46*	14.29 ± 1.18	14.15 ± 1.20	‡ 14.19 ± 1.25	14.26 ± 1.37	14.22 ± 0.18			
Hematocrit	Pork	*23*	42.83 ± 3.31	42.84 ± 3.37	42.84 ± 3.68	43.23 ± 3.79	42.93 ± 0.70	T	0.741	0.008
(%)	Plant	*23*	43.27 ± 3.32	43.04 ± 3.64	42.71 ± 3.53	42.76 ± 3.76	42.94 ± 0.70	T x T	0.384	0.022
	Total	*46*	43.05 ± 3.29	42.94 ± 3.47	42.77 ± 3.57	43.00 ± 3.74	42.94 ± 0.49			
Mean Corpuscular Volume	Pork	*23*	91.17 ± 3.37	91.43 ± 3.09	91.06 ± 3.27	90.97 ± 3.45	91.16 ± 0.67	T	0.859	0.004
(fL)	Plant	*23*	91.43 ± 3.53	91.02 ± 3.52	91.61 ± 3.21	91.42 ± 3.25	91.37 ± 0.67	T x T	0.103	0.048
	Total	*46*	91.30 ± 3.42	91.22 ± 3.28	91.33 ± 3.22	91.20 ± 3.33	91.26 ± 0.48			
Mean Corpuscular Hemoglobin	Pork	*23*	30.35 ± 1.26	30.32 ± 1.28	30.22 ± 1.36	30.28 ± 1.47	30.29 ± 0.28	T	0.281	0.028
(pg)	Plant	*23*	30.25 ± 1.57	29.95 ± 1.34	† 30.37 ± 1.47	30.25 ± 1.31	‡ 30.20 ± 0.28	T x T	0.072	0.052
	Total	*46*	30.30 ± 1.41	30.13 ± 1.31	30.30 ± 1.40	30.26 ± 1.38	30.25 ± 0.20			
Mean Corpuscular Hemoglobin Concentration	Pork	*23*	33.29 ± 0.70	33.16 ± 0.70	33.19 ± 0.87	33.28 ± 0.91	33.23 ± 0.14	T	0.366	0.024
(g/dL)	Plant	*23*	33.10 ± 0.91	32.91 ± 0.69	33.13 ± 0.80	33.07 ± 0.80	33.05 ± 0.14	T x T	0.806	0.007
	Total	*46*	33.20 ± 0.81	33.03 ± 0.69	‡ 33.16 ± 0.83	33.18 ± 0.85	33.14 ± 0.10			
Red Blood Cell Distribution Width	Pork	*23*	12.39 ± 0.52	12.42 ± 0.57	12.35 ± 0.52	12.35 ± 0.52	12.38 ± 0.11	T	0.215	0.033
(%)	Plant	*23*	12.38 ± 0.50	12.48 ± 0.51	‡ 12.46 ± 0.54	12.46 ± 0.50	12.45 ± 0.11	T x T	0.158	0.039
	Total	*46*	12.39 ± 0.51	12.45 ± 0.53	† 12.41 ± 0.53	12.41 ± 0.51	12.41 ± 0.07			
Neutrophils	Pork	*23*	54.02 ± 10.70	53.95 ± 8.27	55.56 ± 9.00	57.36 ± 10.36	55.22 ± 1.57	T	0.339	0.025
(%)	Plant	*23*	53.44 ± 9.38	56.21 ± 9.24	55.15 ± 9.75	55.56 ± 11.18	55.09 ± 1.57	T x T	0.548	0.015
	Total	*46*	53.73 ± 9.95	55.08 ± 8.75	55.35 ± 9.28	56.46 ± 10.70	55.16 ± 1.11			
Lymphocytes	Pork	*23*	33.40 ± 9.30	33.24 ± 6.94	32.03 ± 7.97	30.65 ± 8.05	32.33 ± 1.35	T	0.341	0.025
(%)	Plant	*23*	34.06 ± 8.55	31.52 ± 8.73	32.30 ± 8.52	32.17 ± 8.99	‡ 32.51 ± 1.35	T x T	0.607	0.012
	Total	*46*	33.73 ± 8.84	32.38 ± 7.84	32.17 ± 8.16	31.41 ± 8.47	32.42 ± 0.95			
Monocytes	Pork	*23*	8.53 ± 1.60	9.07 ± 1.81	‡ 8.66 ± 1.74	8.43 ± 2.36	8.67 ± 0.30	T	0.422	0.020
(%)	Plant	*23*	8.65 ± 1.03	8.74 ± 1.56	8.82 ± 1.48	8.73 ± 1.91	8.73 ± 0.30	T x T	0.502	0.017
	Total	*46*	8.59 ± 1.33	8.90 ± 1.68	8.74 ± 1.60	8.58 ± 2.13	8.70 ± 0.21			
Eosinophils	Pork	*23*	3.20 ± 2.51	2.85 ± 2.13	2.77 ± 2.22	2.68 ± 2.37	‡ 2.87 ± 0.46	T	0.205	0.035
(%)	Plant	*23*	2.93 ± 1.89	2.71 ± 2.57	2.93 ± 2.45	2.72 ± 2.29	2.82 ± 0.46	T x T	0.609	0.013
	Total	*46*	3.07 ± 2.20	2.78 ± 2.34	2.85 ± 2.31	2.70 ± 2.30	‡ 2.85 ± 0.32			
Basophils	Pork	*23*	0.63 ± 0.26	0.61 ± 0.19	0.63 ± 0.25	0.61 ± 0.23	0.62 ± 0.04	T	0.503	0.017
(%)	Plant	*23*	0.67 ± 0.24	0.59 ± 0.26	0.57 ± 0.28	† 0.63 ± 0.23	0.61 ± 0.04	T x T	0.380	0.023
	Total	*46*	0.65 ± 0.25	0.60 ± 0.23	0.60 ± 0.26	0.62 ± 0.23	0.62 ± 0.03			
Immature Granulocytes	Pork	*23*	0.25 ± 0.17	0.28 ± 0.19	‡ 0.35 ± 0.50	0.27 ± 0.10	0.29 ± 0.03	T	0.451	0.016
(%)	Plant	*23*	0.27 ± 0.12	0.23 ± 0.13	0.23 ± 0.11	0.21 ± 0.13	‡ 0.24 ± 0.03	T x T	0.270	0.029
	Total	*46*	0.26 ± 0.15	0.26 ± 0.16	0.29 ± 0.36	0.24 ± 0.11	⁑ 0.26 ± 0.02			
Platelets	Pork	*23*	251 ± 49	257 ± 46	‡ 261 ± 46	† 262 ± 55	† 258 ± 11	T	0.107	0.049
(K/µL)	Plant	*23*	248 ± 65	252 ± 61	249 ± 60	250 ± 59	249 ± 11	T x T	0.229	0.033
	Total	*46*	249 ± 57	254 ± 53	† 255 ± 53	† 256 ± 57	‡ 253 ± 8			
Neutrophils	Pork	*23*	3.80 ± 2.14	3.72 ± 1.40	4.09 ± 1.75	4.14 ± 1.94	3.94 ± 0.25	T	0.606	0.012
(K/µL)	Plant	*23*	3.51 ± 1.10	3.81 ± 1.24	3.59 ± 1.37	3.79 ± 1.61	3.67 ± 0.25	T x T	0.636	0.011
	Total	*46*	3.65 ± 1.69	3.76 ± 1.31	3.84 ± 1.58	3.97 ± 1.77	3.81 ± 0.18			
Lymphocytes	Pork	*23*	2.09 ± 0.45	2.17 ± 0.41	2.20 ± 0.42	2.03 ± 0.40	2.12 ± 0.08	T	0.497	0.017
(K/µL)	Plant	*23*	2.16 ± 0.52	2.08 ± 0.60	2.00 ± 0.51	2.06 ± 0.50	2.07 ± 0.08	T x T	0.162	0.039
	Total	*46*	2.13 ± 0.48	2.12 ± 0.51	2.10 ± 0.48	2.04 ± 0.45	2.10 ± 0.06			
Monocytes	Pork	*23*	0.557 ± 0.142	0.599 ± 0.141	‡ 0.609 ± 0.150	† 0.566 ± 0.161	0.583 ± 0.024	T	0.132	0.043
(K/µL)	Plant	*23*	0.558 ± 0.091	0.586 ± 0.148	0.554 ± 0.113	0.564 ± 0.119	0.566 ± 0.024	T x T	0.279	0.029
	Total	*46*	0.558 ± 0.118	0.593 ± 0.143	‡ 0.582 ± 0.134	0.565 ± 0.140	0.574 ± 0.017			
Eosinophils	Pork	*23*	0.201 ± 0.149	0.186 ± 0.145	0.189 ± 0.163	0.172 ± 0.148	‡ 0.187 ± 0.031	T	0.143	0.041
(K/µL)	Plant	*23*	0.187 ± 0.129	0.179 ± 0.173	0.179 ± 0.161	0.170 ± 0.144	0.179 ± 0.031	T x T	0.935	0.003
	Total	*46*	0.194 ± 0.138	0.182 ± 0.158	0.184 ± 0.160	0.171 ± 0.144	† 0.183 ± 0.022			
Basophils	Pork	*23*	0.039 ± 0.015	0.041 ± 0.015	0.044 ± 0.016	† 0.040 ± 0.015	0.041 ± 0.003	T	0.999	0.000
(K/µL)	Plant	*23*	0.042 ± 0.016	0.040 ± 0.016	0.037 ± 0.019	† 0.041 ± 0.015	0.040 ± 0.003	T x T	0.018	0.073
	Total	*46*	0.041 ± 0.016	0.041 ± 0.015	0.041 ± 0.018	0.041 ± 0.015	0.041 ± 0.002			
Immature Granulocytes	Pork	*23*	0.017 ± 0.013	0.019 ± 0.012	0.025 ± 0.035	‡ 0.018 ± 0.008	0.020 ± 0.002	T	0.352	0.022
(K/µL)	Plant	*23*	0.017 ± 0.009	0.017 ± 0.009	0.016 ± 0.008	0.014 ± 0.009	0.016 ± 0.002	T x T	0.279	0.028
	Total	*46*	0.017 ± 0.011	0.018 ± 0.011	0.020 ± 0.026	0.016 ± 0.009	0.018 ± 0.002			
Neutrophils : Lymphocyte Ratio	Pork	*23*	1.96 ± 1.42	1.74 ± 0.62	1.90 ± 0.75	2.16 ± 1.33	1.94 ± 0.15	T	0.566	0.013
	Plant	*23*	1.78 ± 0.98	2.04 ± 1.14	1.95 ± 1.05	2.07 ± 1.45	1.96 ± 0.15	T x T	0.613	0.011
	Total	*46*	1.87 ± 1.21	1.89 ± 0.92	1.92 ± 0.90	2.12 ± 1.38	1.95 ± 0.11			

Data are expressed as means ± standard deviations for the the pork and Plant protein treatments. Data were analyzed using a multivariate and univariate General Linear Model with repeated measures. P-levels, with partial ETA squared (η_p^2) effect size, were reported. General Linear Model analysis revealed no significant overall Wilk's Lambda for Time ($p = 0.137$, $\eta_p^2 = 0.186$) and no significant Treatment x Time ($p = 0.225$, $\eta_p^2 = 0.176$) effect. Greenhouse-Geisser univariate p-levels are listed for time (T) and treatment x time (T x T) interaction effects. Significance was determined via pairwise comparison, with LSD posthoc adjustment, indicated as differences from baseline: † = $p < 0.05$ [‡ = $p > 0.05$ to $p < 0.10$]. η_p^2 effect size values of 0.01 - 0.05 = small, 0.06 - 0.13 = medium, and >0.14 = large.

Table S11. Serum renal function and electrolyte results.

Variable	Treatmen	*n*	Baseline	24-hour	48-hour	72-hour	Mean (SEM)	Effect	p-Value	η_p^2
					Time Point					
Glucose	Pork	*23*	90.4 ± 5.8	89.9 ± 7.1	88.8 ± 7.0	93.3 ± 11.2	90.6 ± 1.4	T	0.034	0.079
(mg/dL)	Plant	*23*	90.4 ± 6.6	90.6 ± 6.1	87.9 ± 5.7 †	91.2 ± 13.0	90.0 ± 1.4	G x T	0.554	0.012
	Total	*46*	90.4 ± 6.2	90.2 ± 6.6	88.3 ± 6.3 †	92.2 ± 12.0	90.3 ± 1.0			
Blood Urea Nitrogen	Pork	*23*	13.3 ± 2.8	14.3 ± 2.7 ‡	14.2 ± 2.6 ‡	14.0 ± 1.7	13.9 ± 0.4	T	0.186	0.037
(mg/dL)	Plant	*23*	13.4 ± 3.6	13.7 ± 2.3	12.9 ± 2.1	12.7 ± 1.8	13.2 ± 0.4	G x T	0.131	0.044
	Total	*46*	13.3 ± 3.2	14.0 ± 2.5 ‡	13.5 ± 2.4 ⁑	13.3 ± 1.8 *	13.6 ± 0.3			
Creatinine	Pork	*23*	1.06 ± 0.13	1.09 ± 0.16 †	1.07 ± 0.14	1.05 ± 0.15	1.07 ± ###	T	0.024	0.073
(mg/dL)	Plant	*23*	1.02 ± 0.11	1.01 ± 0.13	1.01 ± 0.13	0.99 ± 0.13 ‡	1.01 ± ###	G x T	0.193	0.036
	Total	*46*	1.04 ± 0.12	1.05 ± 0.15 ⁑	1.04 ± 0.13	1.02 ± 0.14 †	1.04 ± ###			
Estimated Glomerular Filtration Rate	Pork	*23*	87.2 ± 29.7	84.3 ± 29.6 †	86.1 ± 29.8	88.5 ± 30.0	86.5 ± 4.7	T	0.016	0.080
(ml/min/1.73)	Plant	*23*	100.3 ± 11.5	101.8 ± 12.2	101.0 ± 12.6	104.3 ± 12.4 †	101.9 ± 4.7	G x T	0.236	0.032
	Total	*46*	93.7 ± 23.2 ⁑	93.0 ± 24.1 *	93.5 ± 23.9 *	96.4 ± 24.1 †*	94.2 ± 3.3 *			
BUN/Creatinine Ratio	Pork	*23*	12.7 ± 2.9	13.1 ± 2.4	13.5 ± 2.0	13.4 ± 1.6	13.2 ± 0.4	T	0.580	0.013
	Plant	*23*	13.2 ± 3.7	13.7 ± 2.3	12.8 ± 2.3	13.0 ± 1.8	13.2 ± 0.4	G x T	0.211	0.035
	Total	*46*	12.9 ± 3.3	13.4 ± 2.3	13.2 ± 2.2	13.2 ± 1.7	13.2 ± 0.3			
Sodium	Pork	*23*	140.3 ± 1.9	140.1 ± 1.3	140.2 ± 1.2	139.7 ± 1.4	140.1 ± 0.2	T	0.510	0.017
(meq/L)	Plant	*23*	140.3 ± 1.8	140.1 ± 1.6	140.3 ± 0.8	140.1 ± 1.3	140.2 ± 0.2	G x T	0.758	0.009
	Total	*46*	140.3 ± 1.8	140.1 ± 1.5	140.2 ± 1.0	139.9 ± 1.4	140.1 ± 0.1			
Potassium	Pork	*23*	4.1 ± 0.3	4.0 ± 0.2 †	4.1 ± 0.2	4.3 ± 0.3 †	4.1 ± 0.0	T	0.000	0.241
(meq/L)	Plant	*23*	4.2 ± 0.2	4.0 ± 0.2 †	4.1 ± 0.2	4.2 ± 0.2	4.1 ± 0.0	G x T	0.488	0.018
	Total	*46*	4.2 ± 0.2	4.0 ± 0.2 †	4.1 ± 0.2	4.2 ± 0.3 †	4.1 ± 0.0			
Chloride	Pork	*23*	102.7 ± 1.6	102.8 ± 2.0	103.0 ± 1.4	102.8 ± 1.8	102.8 ± 0.3	T	0.459	0.018
(meq/L)	Plant	*23*	102.9 ± 2.2	103.4 ± 1.8	103.2 ± 2.0	103.5 ± 2.2	103.3 ± 0.3	G x T	0.714	0.009
	Total	*46*	102.8 ± 1.9	103.1 ± 1.9	103.1 ± 1.7	103.2 ± 2.0	103.0 ± 0.2			
Carbon Dioxide	Pork	*23*	24.8 ± 1.8	23.9 ± 1.6 †	23.8 ± 2.1 †	24.2 ± 1.9	24.2 ± 0.3	T	0.011	0.084
(meq/L)	Plant	*23*	24.7 ± 1.9	23.8 ± 2.2 †	24.5 ± 1.9	24.3 ± 2.2	24.3 ± 0.3	G x T	0.304	0.027
	Total	*46*	24.7 ± 1.8	23.8 ± 1.9 †	24.1 ± 2.0 †	24.2 ± 2.0 ‡	24.2 ± 0.2			
Calcium	Pork	*23*	9.5 ± 0.3	9.3 ± 0.3 †	9.4 ± 0.4 †	9.5 ± 0.3	9.4 ± 0.1	T	0.000	0.157
(mg/dL)	Plant	*23*	9.5 ± 0.3	9.3 ± 0.2 †	9.4 ± 0.3	9.4 ± 0.3	9.4 ± 0.1	G x T	0.765	0.008
	Total	*46*	9.5 ± 0.3	9.3 ± 0.3 †	9.4 ± 0.3 †	9.4 ± 0.3	9.4 ± 0.0			

Data are expressed as means ± standard deviations for the the pork and Plant protein treatments. Data were analyzed using a multivariate and univariate General Linear Model with repeated measures. P-levels, with partial ETA squared (η_p^2) effect size, were reported. General Linear Model analysis revealed a significant overall Wilk's Lambda for Time ($p < 0.001$, $\eta_p^2 = 0.188$) and no significant Treatment x Time ($p = 0.833$, $\eta_p^2 = 0.057$) effect. Greenhouse-Geisser univariate p-levels are listed for time (T) and treatment x time (T x T) interaction effects. Significance was determined via pairwise comparison, with LSD posthoc adjustment, indicated as differences from baseline: † = $p < 0.05$ [‡ = $p > 0.05$ to $p < 0.10$]. η_p^2 effect size values of 0.01 - 0.05 = small, 0.06 - 0.13 = medium, and >0.14 = large.

Table S12. Serum blood lipid results.

Variable	Treatment	*n*	Baseline	24-hour	48-hour	72-hour	Mean (SEM)	Effect	p-Value	η_p^2
					Time Point					
Total Cholesterol (mg/dL)	Pork	*23*	146.5 ± 14.5	145.3 ± 13.5	147.5 ± 15.6	151.0 ± 17.9	147.6 ± 3.1	T	0.044	0.069
	Plant	*23*	146.7 ± 18.7	141.8 ± 15.8 †	137.5 ± 14.5 †	137.1 ± 15.6 †	140.8 ± 3.1	T x T	0.000	0.194
	Total	*46*	146.6 ± 16.5	143.6 ± 14.6 †	142.5 ± 15.7 †*	144.0 ± 18.0 *	144.2 ± 2.2			
Triglycerides (mg/dL)	Pork	*23*	80.3 ± 24.3	76.3 ± 29.4	83.7 ± 27.7	76.0 ± 19.0	79.1 ± 4.1	T	0.428	0.020
	Plant	*23*	80.9 ± 29.6	82.1 ± 24.3	83.2 ± 23.2	79.0 ± 21.0	81.3 ± 4.1	T x T	0.812	0.007
	Total	*46*	80.6 ± 26.8	79.2 ± 26.8	83.4 ± 25.2	77.5 ± 19.9	80.2 ± 2.9			
HDL Cholesterol (mg/dL)	Pork	*23*	47.5 ± 9.0	51.2 ± 19.6	47.3 ± 9.0	49.2 ± 9.0 †	48.8 ± 1.8	T	0.351	0.021
	Plant	*23*	50.3 ± 8.7	49.1 ± 8.1	48.1 ± 7.3 †	49.3 ± 8.5	49.2 ± 1.8	T x T	0.343	0.022
	Total	*46*	48.9 ± 8.9	50.2 ± 14.8	47.7 ± 8.1 †	49.3 ± 8.7	49.0 ± 1.3			
LDL Cholesterol (mg/dL)	Pork	*23*	82.4 ± 16.3	81.2 ± 16.1	83.0 ± 16.3	85.5 ± 18.0	83.0 ± 3.3	T	0.110	0.048
	Plant	*23*	78.8 ± 17.6	76.2 ± 16.0	72.6 ± 15.9 †	71.5 ± 16.1 †	74.8 ± 3.3	T x T	0.000	0.170
	Total	*46*	80.6 ± 16.8	78.7 ± 16.1	77.8 ± 16.8 †*	78.5 ± 18.3 *	78.9 ± 2.3			
Non-HDL Cholesterol (mg/dL)	Pork	*23*	99.0 ± 17.6	97.9 ± 16.5	100.2 ± 18.1	97.4 ± 26.6	98.6 ± 3.6	T	0.091	0.056
	Plant	*23*	96.3 ± 18.8	92.7 ± 17.8 †	89.4 ± 17.0 †	87.8 ± 17.5 †	91.6 ± 3.6	T x T	0.136	0.046
	Total	*46*	97.7 ± 18.1	95.3 ± 17.2 ‡	94.8 ± 18.2 †*	92.6 ± 22.8 †	95.1 ± 2.6			
VLDL Cholesterol (mg/dL)	Pork	*23*	16.7 ± 3.2	18.7 ± 12.5	17.2 ± 3.6	16.3 ± 2.5	17.2 ± 0.6	T	0.473	0.014
	Plant	*23*	16.6 ± 3.7	16.6 ± 3.1	16.8 ± 2.7	16.3 ± 2.7	16.6 ± 0.6	T x T	0.541	0.011
	Total	*46*	16.6 ± 3.4	17.7 ± 9.1	17.0 ± 3.1	16.3 ± 2.6	16.9 ± 0.4			
LDL/HDL Ratio	Pork	*23*	1.83 ± 0.64	1.81 ± 0.60	1.84 ± 0.62	1.81 ± 0.57	1.82 ± ###	T	0.044	0.066
	Plant	*23*	1.64 ± 0.49	1.62 ± 0.50	1.57 ± 0.51 ‡	1.51 ± 0.51 †	1.58 ± ###	T x T	0.077	0.055
	Total	*46*	1.74 ± 0.57	1.71 ± 0.56	1.71 ± 0.58	1.66 ± 0.56 †⁑	1.70 ± ###			
Total Cholesterol/HDL Ratio	Pork	*23*	3.20 ± 0.76	3.17 ± 0.70	3.23 ± 0.73	3.15 ± 0.63	3.19 ± ###	T	0.051	0.062
	Plant	*23*	2.99 ± 0.61	2.97 ± 0.60	2.93 ± 0.59	2.86 ± 0.59 †	2.93 ± ###	T x T	0.285	0.028
	Total	*46*	3.10 ± 0.69	3.07 ± 0.65	3.08 ± 0.67	3.01 ± 0.63 †	3.06 ± ###			

Data are expressed as means ± standard deviations for the the pork and Plant protein treatments. Data were analyzed using a multivariate and univariate General Linear Model with repeated measures. P-levels, with partial ETA squared (η_p^2) effect size, were reported. General Linear Model analysis revealed no significant overall Wilk's Lambda for Time (p =0.066, η_p^2=0.087) and a significant Treatment x Time (p=0.013, η_p^2=0.103) effect. Greenhouse-Geisser univariate p-levels are listed for time (T) and treatment x time (T x T) interaction effects. Significance was determined via pairwise comparison, with LSD posthoc adjustment, indicated as differences from baseline: † = p < 0.05 [‡ = p > 0.05 to p < 0.10]. η_p^2 effect size values of 0.01 - 0.05 = small, 0.06 - 0.13 = medium, and >0.14 = large.

Table S13. Markers of protein degradation and muscle and liver enzymes.

Variable	Treatment	*n*	Baseline	24-hour	48-hour	72-hour	Mean (SEM)	Effect	p-Value	η_p^2
					Time Point					
Total Proteins	Pork	*23*	6.79 ± 0.32	6.82 ± 0.25	6.82 ± 0.28	6.88 ± 0.35	6.83 ± 0.06	T	0.457	0.019
(g/dL)	Plant	*23*	6.79 ± 0.37	6.76 ± 0.39	6.74 ± 0.35	6.79 ± 0.38	6.77 ± 0.06	T x T	0.653	0.011
	Total	*46*	6.79 ± 0.34	6.79 ± 0.32	6.78 ± 0.31	6.84 ± 0.36	6.80 ± 0.04			
Albumin	Pork	*23*	4.52 ± 0.16	4.54 ± 0.16	4.49 ± 0.20	4.59 ± 0.22	4.53 ± 0.03	T	0.041	0.063
(g/dL)	Plant	*23*	4.51 ± 0.23	4.55 ± 0.19	4.54 ± 0.18	4.59 ± 0.24 ‡	4.55 ± 0.03	T x T	0.733	0.009
	Total	*46*	4.51 ± 0.19	4.54 ± 0.17	4.52 ± 0.19	4.59 ± 0.22 †	4.54 ± 0.02			
Globulin	Pork	*23*	2.27 ± 0.31	2.28 ± 0.29	2.32 ± 0.24	2.29 ± 0.28	2.29 ± 0.06	T	0.750	0.008
(g/dL)	Plant	*23*	2.28 ± 0.34	2.21 ± 0.33 ‡	2.20 ± 0.30	2.20 ± 0.29 ‡	2.22 ± 0.06	T x T	0.192	0.036
	Total	*46*	2.28 ± 0.32	2.25 ± 0.31	2.26 ± 0.28	2.25 ± 0.29	2.26 ± 0.04			
Albumin-Globulin Ratio	Pork	*23*	2.03 ± 0.30	2.02 ± 0.28	1.96 ± 0.25	2.04 ± 0.29	2.01 ± 0.05	T	0.306	0.027
	Plant	*23*	2.02 ± 0.29	2.09 ± 0.31	2.10 ± 0.29 ‡	2.12 ± 0.30 †	2.08 ± 0.05	T x T	0.189	0.036
	Total	*46*	2.02 ± 0.30	2.05 ± 0.29	2.03 ± 0.28 ⁑	2.08 ± 0.30 ‡	2.05 ± 0.04			
Total Bilirubin	Pork	*23*	0.56 ± 0.31	0.41 ± 0.17 †	0.39 ± 0.16 †	0.45 ± 0.23 ‡	0.45 ± 0.04	T	0.025	0.082
(mg/dL)	Plant	*23*	0.57 ± 0.35	0.53 ± 0.20	0.56 ± 0.18	0.51 ± 0.20	0.54 ± 0.04	T x T	0.113	0.049
	Total	*46*	0.56 ± 0.33	0.47 ± 0.19 †	0.48 ± 0.19 †	0.48 ± 0.21 †	0.50 ± 0.03			
Alkaline Phosphatase	Pork	*23*	77.5 ± 17.6	76.5 ± 16.1	76.0 ± 17.1 ‡	77.4 ± 17.3	76.9 ± 3.4	T	0.014	0.083
(U/L)	Plant	*23*	76.4 ± 16.1	75.4 ± 15.6	74.6 ± 15.7 †	76.6 ± 15.9	75.8 ± 3.4	T x T	0.945	0.002
	Total	*46*	77.0 ± 16.7	76.0 ± 15.7 ‡	75.3 ± 16.2 †	77.0 ± 16.4	76.3 ± 2.4			
Aspartate Aminotransferase	Pork	*23*	23.3 ± 9.0	25.9 ± 9.3 †	23.6 ± 7.7	22.9 ± 8.7	23.9 ± 1.5	T	0.027	0.082
(U/L)	Plant	*23*	22.2 ± 9.0	23.3 ± 7.3	22.1 ± 5.1	21.3 ± 5.5	22.2 ± 1.5	T x T	0.697	0.007
	Total	*46*	22.7 ± 8.9	24.6 ± 8.4 †*	22.9 ± 6.5 *	22.1 ± 7.2	23.1 ± 1.1			
Alanine Aminotransferase	Pork	*23*	19.9 ± 11.9	19.9 ± 9.5	19.6 ± 9.1	19.3 ± 8.9	19.7 ± 2.0	T	0.641	0.009
(U/L)	Plant	*23*	19.6 ± 11.5	19.5 ± 9.8	19.1 ± 9.2	19.2 ± 8.7	19.3 ± 2.0	T x T	0.933	0.001
	Total	*46*	19.7 ± 11.5	19.7 ± 9.6	19.3 ± 9.1	19.2 ± 8.7	19.5 ± 1.4			
Lactate Dehydrogenase	Pork	*23*	177 ± 24	180 ± 27	173 ± 24	169 ± 23	175 ± 6	T	0.036	0.079
(U/L)	Plant	*23*	188 ± 59	181 ± 32	172 ± 30 †	175 ± 32 †	179 ± 6	T x T	0.348	0.023
	Total	*46*	182 ± 45	181 ± 30	173 ± 27 ‡	172 ± 28 †	177 ± 4			
Creatine Kinase	Pork	*23*	235 ± 161	397 ± 331 †	288 ± 228	278 ± 371	299 ± 49	T	0.006	0.120
(U/L)	Plant	*23*	275 ± 399	337 ± 280	232 ± 165	194 ± 122	260 ± 49	T x T	0.280	0.028
	Total	*46*	255 ± 301	367 ± 304 †	260 ± 199	236 ± 276	280 ± 35			

Data are expressed as means ± standard deviations for the Pork and Plant protein treatments. Data were analyzed using a multivariate and univariate General Linear Model with repeated measures. P-levels, with partial ETA squared (η_p^2) effect size, were reported. General Linear Model analysis revealed no significant overall Wilk's Lambda for Time ($p < 0.001$, $\eta_p^2 = 0.153$) and no significant Treatment x Time ($p = 0.642$, $\eta_p^2 = 0.060$) effect. Greenhouse-Geisser univariate p-levels are listed for time (T) and treatment x time (T x T) interaction effects. Significance was determined via pairwise comparison, with LSD posthoc adjustment, indicated as differences from baseline: † = $p < 0.05$ [‡ = $p > 0.05$ to $p < 0.10$]. η_p^2 effect size values of 0.01 - 0.05 = small, 0.06 - 0.13 = medium, and >0.14 = large.

Table S14. Hormonal markers of catabolism and anabolism.

Variable	Treatment	*n*	Baseline	24-hour	48-hour	72-hour	Mean (SEM)	Effect	p-Value	η_p^2
					Time Point					
Cortisol	Pork	*23*	18.68 ± 3.00	17.51 ± 4.33	15.87 ± 4.88 †	15.70 ± 4.74 †	16.94 ± 0.68	T	0.005	0.096
(mcg/dL)	Plant	*23*	19.15 ± 4.48	18.49 ± 4.38	18.15 ± 4.17	16.95 ± 5.51 ‡	18.18 ± 0.68	T x T	0.650	0.012
	Total	*46*	18.91 ± 3.78	18.00 ± 4.33	17.01 ± 4.64 †⁑	16.32 ± 5.12 †	17.56 ± 0.48			
Testosterone	Pork	*23*	6.75 ± 3.96	6.35 ± 3.45 ‡	6.13 ± 3.43 †	6.43 ± 3.60	6.42 ± 0.68	T	0.135	0.042
(ng/dL)	Plant	*23*	6.62 ± 3.06	6.38 ± 2.74	6.41 ± 3.17	6.25 ± 3.12	6.41 ± 0.68	T x T	0.590	0.014
	Total	*46*	6.69 ± 3.50	6.36 ± 3.08 †	6.27 ± 3.27 ‡	6.34 ± 3.34	6.42 ± 0.48			
Testosterone/Cortisol Ratio	Pork	*23*	0.37 ± 0.21	0.39 ± 0.23	0.50 ± 0.52 ‡	0.50 ± 0.47	0.44 ± 0.06	T	0.104	0.052
	Plant	*23*	0.36 ± 0.20	0.36 ± 0.18	0.37 ± 0.20	0.52 ± 0.65	0.40 ± 0.06	T x T	0.501	0.014
	Total	*46*	0.37 ± 0.21	0.38 ± 0.20	0.43 ± 0.39	0.51 ± 0.56 ‡	0.42 ± 0.04			

Data are expressed as means ± standard deviations for the pork and plant protein treatments. Data were analyzed using a multivariate and univariate General Linear Model with repeated measures. P-levels, with partial ETA squared (η_p^2) effect size, were reported. General Linear Model analysis revealed a significant overall Wilk's Lambda for Time ($p = 0.038$, $\eta_p^2 = 0.044$) but no significant Treatment x Time effects ($p = 0.894$, $\eta_p^2 = 0.011$). Greenhouse-Geisser univariate p-levels are listed for time (T) and treatment x time (T x T) interaction effects. Significance was determined via pairwise comparison, with LSD posthoc adjustment, indicated as differences from baseline: † = $p < 0.05$ [‡ = $p > 0.05$ to $p < 0.10$]. η_p^2 effect size values of 0.01 - 0.05 = small, 0.06 - 0.13 = medium, and >0.14 = large.

Table S15. Inflammatory markers.

Variable	Treatment	*n*	Baseline	24-hour	48-hour	72-hour	Mean (SEM)	Effect	p-Value	η_p^2
					Time Point					
IL-1β	Pork	*23*	0.76 ± 0.17	0.79 ± 0.23	0.77 ± 0.20	0.74 ± 0.16	0.77 ± ###	T	0.219	0.033
(pg/mL)	Plant	*23*	0.78 ± 0.28	0.82 ± 0.35	0.81 ± 0.23	0.79 ± 0.28	0.80 ± ###	G x T	0.900	0.004
	Total	*46*	0.77 ± 0.23	0.80 ± 0.30 ‡	0.79 ± 0.21	0.77 ± 0.23	0.78 ± ###			
IL-10	Pork	*23*	7.31 ± 2.73	7.16 ± 2.88	7.28 ± 2.87	7.15 ± 2.46	7.23 ± ###	T	0.954	0.002
(pg/mL)	Plant	*23*	7.34 ± 2.71	7.57 ± 3.10	7.23 ± 2.87	7.41 ± 2.94	7.39 ± ###	G x T	0.727	0.009
	Total	*46*	7.32 ± 2.69	7.36 ± 2.97	7.25 ± 2.84	7.28 ± 2.68	7.31 ± ###			
IL-6	Pork	*23*	22.7 ± 13.9	23.5 ± 13.8	22.9 ± 13.9	23.0 ± 14.0	23.0 ± 2.9	T	0.325	0.026
(pg/mL)	Plant	*23*	25.0 ± 15.2	25.5 ± 17.4	24.8 ± 15.5	22.2 ± 13.9	24.4 ± 2.9	G x T	0.377	0.022
	Total	*46*	23.8 ± 14.4	24.5 ± 15.6	23.9 ± 14.6	22.6 ± 13.8	23.7 ± 2.1			
GM-CSF	Pork	*23*	4.24 ± 1.98	4.46 ± 1.92	4.34 ± 1.92	4.28 ± 1.96	4.33 ± ###	T	0.103	0.047
(pg/mL)	Plant	*23*	4.33 ± 2.09	4.33 ± 2.19	4.46 ± 2.23	3.99 ± 2.05 ‡	4.28 ± ###	G x T	0.270	0.029
	Total	*46*	4.29 ± 2.01	4.40 ± 2.04	4.40 ± 2.06	4.14 ± 1.99	4.31 ± ###			
IL-5	Pork	*23*	5.21 ± 1.41	5.21 ± 1.31	5.42 ± 1.28	5.23 ± 0.91	5.27 ± ###	T	0.587	0.013
(pg/mL)	Plant	*23*	5.30 ± 1.10	5.31 ± 1.49	5.28 ± 1.02	5.18 ± 1.48	5.27 ± ###	G x T	0.629	0.012
	Total	*46*	5.25 ± 1.25	5.26 ± 1.39	5.35 ± 1.15	5.21 ± 1.21	5.27 ± ###			
IFN-γ	Pork	*23*	5.05 ± 1.59	5.16 ± 1.13	5.08 ± 1.14	5.09 ± 1.08	5.10 ± ###	T	0.636	0.011
(pg/mL)	Plant	*23*	5.26 ± 1.36	5.36 ± 1.91	5.06 ± 1.16	5.32 ± 1.51	5.25 ± ###	G x T	0.805	0.006
	Total	*46*	5.16 ± 1.47	5.26 ± 1.55	5.07 ± 1.14	5.21 ± 1.30	5.17 ± ###			
TNF-α	Pork	*23*	6.39 ± 1.90	6.54 ± 1.91	6.69 ± 1.98	6.19 ± 1.60	6.45 ± ###	T	0.286	0.028
(pg/mL)	Plant	*23*	6.52 ± 1.87	6.67 ± 2.33	6.61 ± 2.18	6.45 ± 2.24	6.56 ± ###	G x T	0.813	0.006
	Total	*46*	6.46 ± 1.87	6.60 ± 2.11	6.65 ± 2.06	6.32 ± 1.93	6.51 ± ###			
IL-2	Pork	*23*	18.2 ± 7.8	18.4 ± 7.2	19.3 ± 10.6	18.9 ± 7.7	18.7 ± 2.6	T	0.215	0.035
(pg/mL)	Plant	*23*	19.7 ± 13.2	24.0 ± 25.9 †	17.4 ± 7.7	21.1 ± 17.4	20.6 ± 2.6	G x T	0.117	0.052
	Total	*46*	19.0 ± 10.8	21.2 ± 19.0	18.3 ± 9.2	20.0 ± 13.4	19.6 ± 1.8			
IL-4	Pork	*23*	35.4 ± 14.0	34.3 ± 12.5	34.4 ± 12.5	34.8 ± 12.6	34.7 ± 3.2	T	0.539	0.016
(pg/mL)	Plant	*23*	36.4 ± 18.7	37.0 ± 20.3	35.1 ± 14.7	35.3 ± 19.0	36.0 ± 3.2	G x T	0.568	0.015
	Total	*46*	35.9 ± 16.3	35.7 ± 16.8	34.8 ± 13.4	35.0 ± 15.9	35.3 ± 2.3			
IL-8	Pork	*23*	23.0 ± 8.0	22.7 ± 9.1	20.0 ± 6.7 †	21.0 ± 8.1	21.7 ± 1.5	T	0.250	0.031
(pg/mL)	Plant	*23*	23.4 ± 8.3	23.6 ± 8.2	23.2 ± 8.6	23.6 ± 8.0	23.5 ± 1.5	G x T	0.355	0.024
	Total	*46*	23.2 ± 8.1	23.1 ± 8.6	21.6 ± 7.8 †	22.3 ± 8.1	22.6 ± 1.1			

Data are expressed as means ± standard deviations for the the pork and Plant protein treatments. Data were analyzed using a multivariate and univariate General Linear Model with repeated measures. P-levels, with partial ETA squared (η_p^2) effect size, were reported. General Linear Model analysis revealed no significant overall Wilk's Lambda for Time ($p = 0.238$, $\eta_p^2 = 0.087$) and no significant Treatment x Time ($p = 0.719$, $\eta_p^2 = 0.063$) effect. Greenhouse-Geisser univariate p-levels are listed for time (T) and treatment x time (G x T) interaction effects. Significance was determined via pairwise comparison, with LSD posthoc adjustment, indicated as differences from baseline: † = $p < 0.05$ [‡ = $p > 0.05$ to $p < 0.10$]. η_p^2 effect size values of 0.01 - 0.05 = small, 0.06 - 0.13 = medium, and >0.14 = large.

Table S16. Urinary markers of catabolism.

Variable	Treatment	*n*	Time Point: Baseline	24-hour	48-hour	72-hour	Mean (SEM)	Effect	p-Value	η_p^2
Urea Nitrogen (mg/dL)	Pork	*23*	782 ± 291	797 ± 358	804 ± 387	817 ± 377	800 ± 64	T	0.215	0.034
	Plant	*23*	753 ± 339	871 ± 385	865 ± 493	678 ± 322	792 ± 64	T x T	0.154	0.040
	Total	*46*	767 ± 312	834 ± 370	835 ± 440	747 ± 354	796 ± 45			
Urea Nitrogen (g/d)	Pork	*23*	10.41 ± 4.36	9.01 ± 4.74	9.19 ± 3.21	10.40 ± 3.93	9.75 ± 0.63	T	0.349	0.024
	Plant	*23*	8.30 ± 3.37	9.16 ± 3.51	8.48 ± 3.09	9.16 ± 3.81	8.77 ± 0.63	T x T	0.222	0.033
	Total	*46*	9.36 ± 4.00 ⁑	9.08 ± 4.13	8.84 ± 3.14	9.78 ± 3.88	9.26 ± 0.45			
Creatinine (mg/dL)	Pork	*23*	131.7 ± 50.3	163.2 ± 80.1 †	156.0 ± 79.4	152.4 ± 75.1	150.8 ± 13.4	T	0.007	0.090
	Plant	*23*	134.9 ± 85.2	158.8 ± 68.9 ‡	161.0 ± 99.3	120.2 ± 61.0	143.7 ± 13.4	T x T	0.209	0.034
	Total	*46*	133.3 ± 69.2	161.0 ± 73.9 †	158.5 ± 88.9 †	136.3 ± 69.5	147.3 ± 9.5			
Creatinine (g/d)	Pork	*23*	1.606 ± 0.629	1.743 ± 0.860	1.863 ± 0.669 ‡	1.943 ± 0.810 †	1.789 ± #####	T	0.122	0.044
	Plant	*23*	1.410 ± 0.490	1.635 ± 0.595	1.489 ± 0.582	1.570 ± 0.672	1.526 ± #####	T x T	0.488	0.018
	Total	*46*	1.508 ± 0.566	1.689 ± 0.733	1.676 ± 0.648 ‡*	1.757 ± 0.760 †⁑	1.657 ± ##### ⁑			

Data are expressed as means ± standard deviations for the the pork and Plant protein treatments. Data were analyzed using a multivariate and univariate General Linear Model with repeated measures. P-levels, with partial ETA squared (η_p^2) effect size, were reported. General Linear Model analysis revealed a significant overall Wilk's Lambda for Time ($p < 0.001$, $\eta_p^2 = 0.085$) and a significant Treatment x Time ($p = 0.012$, $\eta_p^2 = 0.063$) effect. Greenhouse-Geisser univariate p-levels are listed for time (T) and treatment x time (T x T) interaction effects. Significance was determined via pairwise comparison, with LSD posthoc adjustment, indicated as differences from baseline: † = $p < 0.05$ [‡ = $p > 0.05$ to $p < 0.10$]. η_p^2 effect size values of 0.01 - 0.05 = small, 0.06 - 0.13 = medium, and >0.14 = large.

Table S17. Diet satisfaction inventory..

Variable	Treatment	*n*	Time Point: Baseline	24-hour	48-hour	72-hour	Mean (SEM)	Effect	p-Value	η_p^2
Appetite	Pork	*23*	4.5 ± 3.3	5.7 ± 2.9 †	6.0 ± 2.2 †	6.5 ± 2.2 †	5.7 ± 0.5	T	0.011	0.086
	Plant	*23*	5.3 ± 3.1	5.2 ± 2.7	5.8 ± 2.4	5.7 ± 3.0	5.5 ± 0.5	T x T	0.132	0.043
	Total	*46*	4.9 ± 3.2	5.4 ± 2.8	5.9 ± 2.3 †	6.1 ± 2.6 †⁑	5.6 ± 0.3			
Hunger	Pork	*23*	3.9 ± 3.5	5.8 ± 3.0 †	5.4 ± 2.9 †	5.9 ± 2.8 †	5.2 ± 0.5	T	0.009	0.086
	Plant	*23*	4.1 ± 3.1	4.3 ± 3.2	5.2 ± 3.1 ‡	4.3 ± 2.9	4.5 ± 0.5	T x T	0.053	0.057
	Total	*46*	4.0 ± 3.3	5.0 ± 3.2 †	5.3 ± 3.0 †	5.1 ± 2.9 †	4.9 ± 0.4			
Satisfaction from Food	Pork	*23*	7.4 ± 1.5	7.8 ± 1.9	7.0 ± 2.6	7.6 ± 2.0	7.4 ± 0.4	T	0.264	0.030
	Plant	*23*	7.1 ± 2.2	7.3 ± 2.2	7.0 ± 2.5	7.1 ± 2.4	7.1 ± 0.4	T x T	0.805	0.007
	Total	*46*	7.3 ± 1.9	7.6 ± 2.0	7.0 ± 2.5	7.3 ± 2.2	7.3 ± 0.3			
Feeling of Fullness	Pork	*23*	7.7 ± 2.2	7.6 ± 1.9	7.9 ± 1.8	7.9 ± 1.7	7.8 ± 0.3	T	0.714	0.008
	Plant	*23*	7.7 ± 2.1	7.9 ± 1.9	7.4 ± 2.3	8.0 ± 1.3	7.7 ± 0.3	T x T	0.489	0.017
	Total	*46*	7.7 ± 2.2	7.7 ± 1.9	7.7 ± 2.0	7.9 ± 1.5	7.8 ± 0.2			
Amount of Energy	Pork	*23*	7.3 ± 1.3	7.0 ± 1.0 ‡	6.8 ± 1.4	7.0 ± 1.5	7.0 ± 0.2	T	0.110	0.045
	Plant	*23*	6.7 ± 1.3	7.1 ± 1.6	6.4 ± 1.6	6.9 ± 1.6	6.8 ± 0.2	T x T	0.289	0.028
	Total	*46*	7.0 ± 1.3	7.1 ± 1.3	6.6 ± 1.5 ‡	7.0 ± 1.5	6.9 ± 0.2			

Data are expressed as means ± standard deviations for the the pork and Plant protein treatments. Data were analyzed using a multivariate and univariate General Linear Model with repeated measures. P-levels, with partial ETA squared (η_p^2) effect size, were reported. General Linear Model analysis revealed a significant overall Wilk's Lambda for Time ($p = 0.015$, η_p^2=0.072) with no significant Treatment x Time (p=0.409, η_p^2=0.039) effect. Greenhouse-Geisser univariate p-levels are listed for time (T) and treatment x time (T x T) interaction effects. Significance was determined via pairwise comparison, with LSD posthoc adjustment, indicated as differences from baseline: † = $p < 0.05$ [‡ = $p > 0.05$ to $p < 0.10$]. η_p^2 effect size values of 0.01 - 0.05 = small, 0.06 - 0.13 = medium, and >0.14 = large.

Table S18. Frequency and Severeity of Side Effects

Symptom	Hours	Group	None	Minimal	Slight	Occasional	Frequent	Severe	χ^2 p-level	p level
			Rating of Symptom Frequency							
Dizziness	0	Pork	21	1	1	0	0	0	1.023	0.600
		Plant	22	1	0	0	0	0		
		Total	43	2	1	0	0	0		
	24	Pork	20	3	0	0	0	0	2.024	0.363
		Plant	21	1	1	0	0	0		
		Total	41	4	1	0	0	0		
	48	Pork	20	2	1	0	0	0	1.429	0.490
		Plant	22	1	0	0	0	0		
		Total	42	3	1	0	0	0		
	72	Pork	21	2	0	0	0	0	3.230	0.221
		Plant	22	0	1	0	0	0		
		Total	43	2	1	0	0	0		
Headache	0	Pork	18	4	0	1	0	0	2.000	0.572
		Plant	18	4	1	0	0	0		
		Total	36	8	1	1	0	0		
	24	Pork	15	3	4	0	1	0	3.425	0.331
		Plant	17	5	1	0	0	0		
		Total	32	8	5	0	1	0		
	48	Pork	15	5	2	1	0	0	1.825	0.610
		Plant	17	3	3	0	0	0		
		Total	32	8	5	1	0	0		
	72	Pork	18	5	0	0	0	0	1.029	0.598
		Plant	17	5	0	1	0	0		
		Total	35	10	0	1	0	0		
Tachycardia	0	Pork	22	0	0	1	0	0	5.220	0.156
		Plant	19	3	1	0	0	0	.	
		Total	41	3	1	1	0	0		
	24	Pork	22	0	0	1	0	0	2.000	0.368
		Plant	22	0	0	0	1	0		
		Total	44	0	0	1	1	0		
	48	Pork	22	0	0	1	0	0	1.000	1.000
		Plant	22	0	0	1	0	0		
		Total	44	0	0	2	0	0		
	72	Pork	22	1	0	0	0	0	0.000	1.000
		Plant	22	1	0	0	0	0		
		Total	44	2	0	0	0	0		
Heart Palpitations	0	Pork	23	0	0	0	0	0		
		Plant	23	0	0	0	0	0		
		Total	46	0	0	0	0	0		
	24	Pork	23	0	0	0	0	0		
		Plant	23	0	0	0	0	0		
		Total	46	0	0	0	0	0		
	48	Pork	23	0	0	0	0	0		
		Plant	23	0	0	0	0	0		
		Total	46	0	0	0	0	0		
	72	Pork	22	1	0	0	0	0	1.022	0.312
		Plant	23	0	0	0	0	0		
		Total	45	1	0	0	0	0		
Dyspnea	0	Pork	21	1	0	1	0	0	2.091	0.352
		Plant	23	0	0	0	0	0		
		Total	44	1	0	1	0	0		
	24	Pork	21	1	1	0	0	0	1.023	0.600
		Plant	22	0	1	0	0	0		
		Total	43	1	2	0	0	0		
	48	Pork	22	0	0	0	1	0	2.000	0.368
		Plant	22	0	1	0	0	0		
		Total	44	0	1	0	1	0		
	72	Pork	22	0	0	0	1	0	0.000	1.000
		Plant	22	0	1	0	0	0		
		Total	44	0	1	0	0	0		
Nervousness	0	Pork	20	2	0	0	1	0	2.105	0.551
		Plant	18	2	2	0	1	0		
		Total	38	4	2	0	2	0		
	24	Pork	20	2	0	0	1	0	1.333	0.721
		Plant	20	1	1	0	1	0		
		Total	40	3	1	0	2	0		
	48	Pork	19	3	0	0	1	0	0.000	1.000
		Plant	19	3	0	0	1	0		
		Total	38	6	0	0	2	0		
	72	Pork	21	2	0	0	0	0	1.300	0.522
		Plant	19	3	0	0	1	0		
		Total	40	5	0	0	1	0		
Blurred Vision	0	Pork	23	0	0	0	0	0		
		Plant	23	0	0	0	0	0		
		Total	46	0	0	0	0	0		
	24	Pork	23	0	0	0	0	0		
		Plant	23	0	0	0	0	0		
		Total	46	0	0	0	0	0		
	48	Pork	23	0	0	0	0	0	1.023	0.312
		Plant	22	1	0	0	0	0		
		Total	45	1	0	0	0	0		
	72	Pork	22	0	1	0	0	0	2.000	0.368
		Plant	22	1	0	0	0	0		
		Total	44	1	1	0	0	0		
Other	0	Pork	23	0	0	0	0	0		
		Plant	23	0	0	0	0	0		
		Total	46	0	0	0	0	0		
	24	Pork	22	0	1	0	0	0	1.022	0.312
		Plant	23	0	0	0	0	0		
		Total	45	0	1	0	0	0		
	48	Pork	23	0	0	0	0	0		
		Plant	22	0	0	0	0	0		
		Total	45	0	0	0	0	0		
	72	Pork	23	0	0	0	0	0		
		Plant	23	0	0	0	0	0		
		Total	46	0	0	0	0	0		

Symptom	Hours	Group	None	Minimal	Slight	Moderate	Severe	Very Severe	χ^2	χ2 p-level
			Rating of Symptom Severity							
Dizziness	0	Pork	21	1	1	0	0	0	1.023	0.600
		Plant	22	1	0	0	0	0		
		Total	43	2	1	0	0	0		
	24	Pork	20	3	0	0	0	0	0.224	0.636
		Plant	21	2	0	0	0	0		
		Total	41	5	0	0	0	0		
	48	Pork	20	2	1	0	0	0	1.429	0.490
		Plant	22	1	0	0	0	0		
		Total	42	3	1	0	0	0		
	72	Pork	21	2	0	0	0	0	3.032	0.221
		Plant	22	0	1	0	0	0		
		Total	43	2	1	0	0	0		
Headache	0	Pork	18	3	1	1	0	0	0.000	1.000
		Plant	18	3	1	1	0	0		
		Total	36	6	2	2	0	0		
	24	Pork	15	2	4	1	1	0	1.992	0.737
		Plant	17	3	2	1	0	0		
		Total	32	5	6	2	1	0		
	48	Pork	15	3	3	1	1	0	2.268	0.687
		Plant	17	4	1	1	0	0		
		Total	32	7	4	2	1	0		
	72	Pork	18	4	1	0	0	0	2.140	0.544
		Plant	17	5	0	1	0	0		
		Total	35	9	1	1	0	0		
Tachycardia	0	Pork	22	1	0	0	0	0	2.553	0.466
		Plant	19	2	1	1	0	0		
		Total	41	3	1	1	0	0		
	24	Pork	22	1	0	0	0	0	0.000	1.000
		Plant	22	1	0	0	0	0		
		Total	44	2	0	0	0	0		
	48	Pork	22	1	0	0	0	0	2.000	0.368
		Plant	22	0	1	0	0	0		
		Total	44	1	1	0	0	0		
	72	Pork	22	1	0	0	0	0	2.000	0.368
		Plant	22	0	1	0	0	0		
		Total	44	1	1	0	0	0		
Heart Palpitations	0	Pork	23	0	0	0	0	0		
		Plant	23	0	0	0	0	0		
		Total	46	0	0	0	0	0		
	24	Pork	23	0	0	0	0	0		
		Plant	23	0	0	0	0	0		
		Total	46	0	0	0	0	0		
	48	Pork	23	0	0	0	0	0		
		Plant	23	0	0	0	0	0		
		Total	46	0	0	0	0	0		
	72	Pork	22	1	0	0	0	0	1.022	0.312
		Plant	22	0	1	0	0	0		
		Total	44	1	1	0	0	0		
Dyspnea	0	Pork	21	2	0	0	0	0	2.091	0.148
		Plant	23	0	0	0	0	0		
		Total	44	2	0	0	0	0		
	24	Pork	21	2	0	0	0	0	0.357	0.550
		Plant	22	1	0	0	0	0		
		Total	43	3	0	0	0	0		
	48	Pork	22	1	0	0	0	0	2.000	0.368
		Plant	22	0	1	0	0	0		
		Total	44	1	1	0	0	0		
	72	Pork	22	1	0	0	0	0	2.000	0.368
		Plant	22	0	1	0	0	0		
		Total	44	1	1	0	0	0		
Nervousness	0	Pork	20	2	1	0	0	0	1.439	0.697
		Plant	18	2	2	1	0	0		
		Total	38	4	3	1	0	0		
	24	Pork	20	2	0	1	0	0	2.000	0.368
		Plant	20	2	1	0	0	0		
		Total	40	4	1	1	0	0		
	48	Pork	19	3	1	0	0	0	2.226	0.527
		Plant	20	2	0	1	0	0		
		Total	39	5	1	1	0	0		
	72	Pork	20	1	2	0	0	0	0.359	0.836
		Plant	19	2	2	0	0	0		
		Total	39	3	4	0	0	0		
Blurred Vision	0	Pork	23	0	0	0	0	0		
		Plant	23	0	0	0	0	0		
		Total	46	0	0	0	0	0		
	24	Pork	23	0	0	0	0	0		
		Plant	23	0	0	0	0	0		
		Total	46	0	0	0	0	0		
	48	Pork	23	0	0	0	0	0	2.091	0.352
		Plant	21	1	1	0	0	0		
		Total	44	1	1	0	0	0		
	72	Pork	23	0	0	0	0	0		
		Plant	23	0	0	0	0	0		
		Total	46	0	0	0	0	0		
Other	0	Pork	23	0	0	0	0	0		
		Plant	23	0	0	0	0	0		
		Total	46	0	0	0	0	0		
	24	Pork	22	1	0	0	0	0	1.022	0.312
		Plant	23	0	0	0	0	0		
		Total	45	1	0	0	0	0		
	48	Pork	23	0	0	0	0	0	1.022	0.312
		Plant	22	0	0	0	1	0		
		Total	45	0	0	0	1	0		
	72	Pork	23	0	0	0	0	0		
		Plant	23	0	0	0	0	0		
		Total	46	0	0	0	0	0		

Data are presented as frequencies. Statistical significance is detailed by chi-squared analysis; $p < 0.05$ considered significant. Totals are included but separate from analysis.

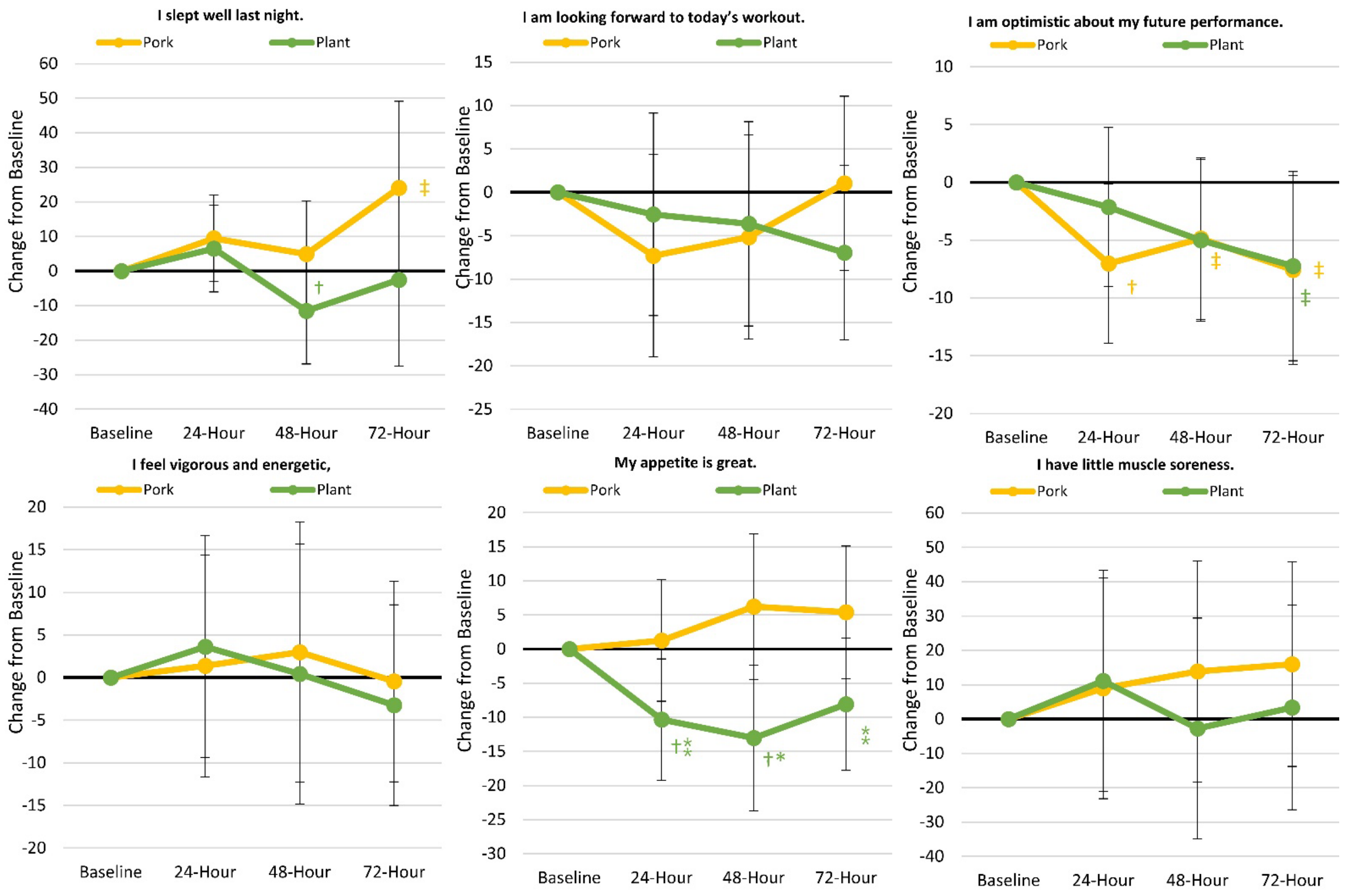

Figure S1. Changes in Readiness to Perform Questionnaire results. Data are displayed as mean percentage changes from baseline with 95% confidence intervals. † = $p < 0.05$ (‡ = $p > 0.05$ to $p < 0.10$) from baseline values. * = $p < 0.05$ (⁑ = $p > 0.05$ to $p < 0.10$) difference between diet treatments.

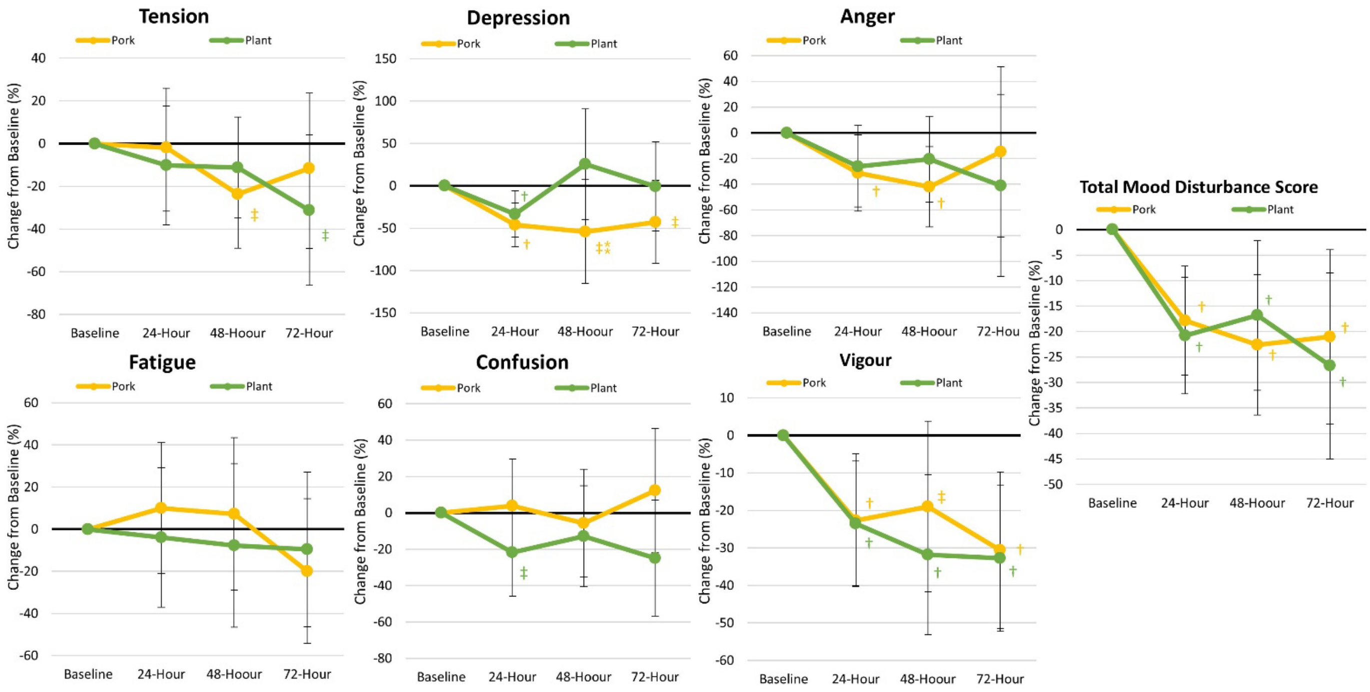

Figure S2. Percent changes in Profile of Mood States categories. Data are displayed as mean percentage changes from baseline with 95% confidence intervals. † = $p < 0.05$ (‡ = $p > 0.05$ to $p < 0.10$) from baseline values. ⁑ = $p > 0.05$ to $p < 0.10$ difference between diet treatments.

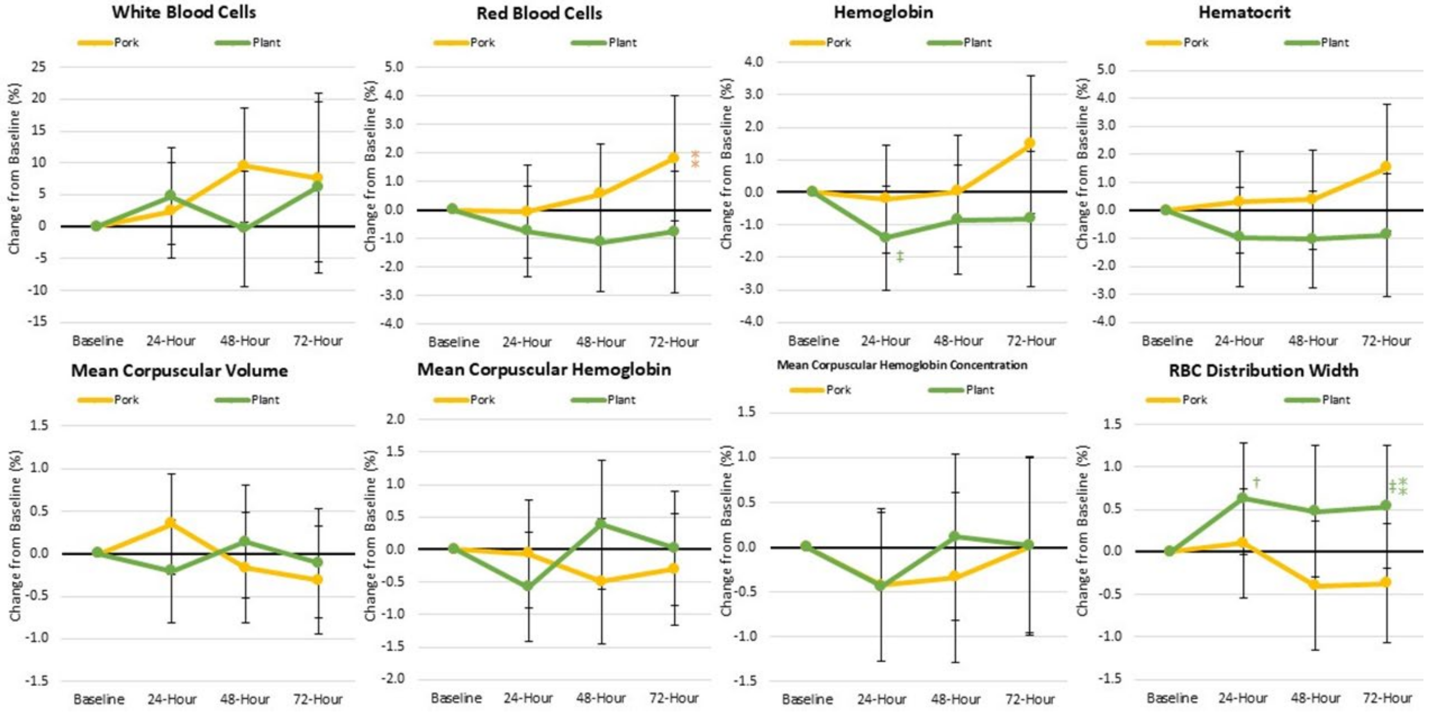

Figure S3a. Percent changes in white and red cells. Data are displayed as mean percentage changes from baseline with 95% confidence intervals. † = $p < 0.05$ (‡ = $p > 0.05$ to $p < 0.10$) from baseline values. ⁑ = $p > 0.05$ to $p < 0.10$ difference between diet treatments.

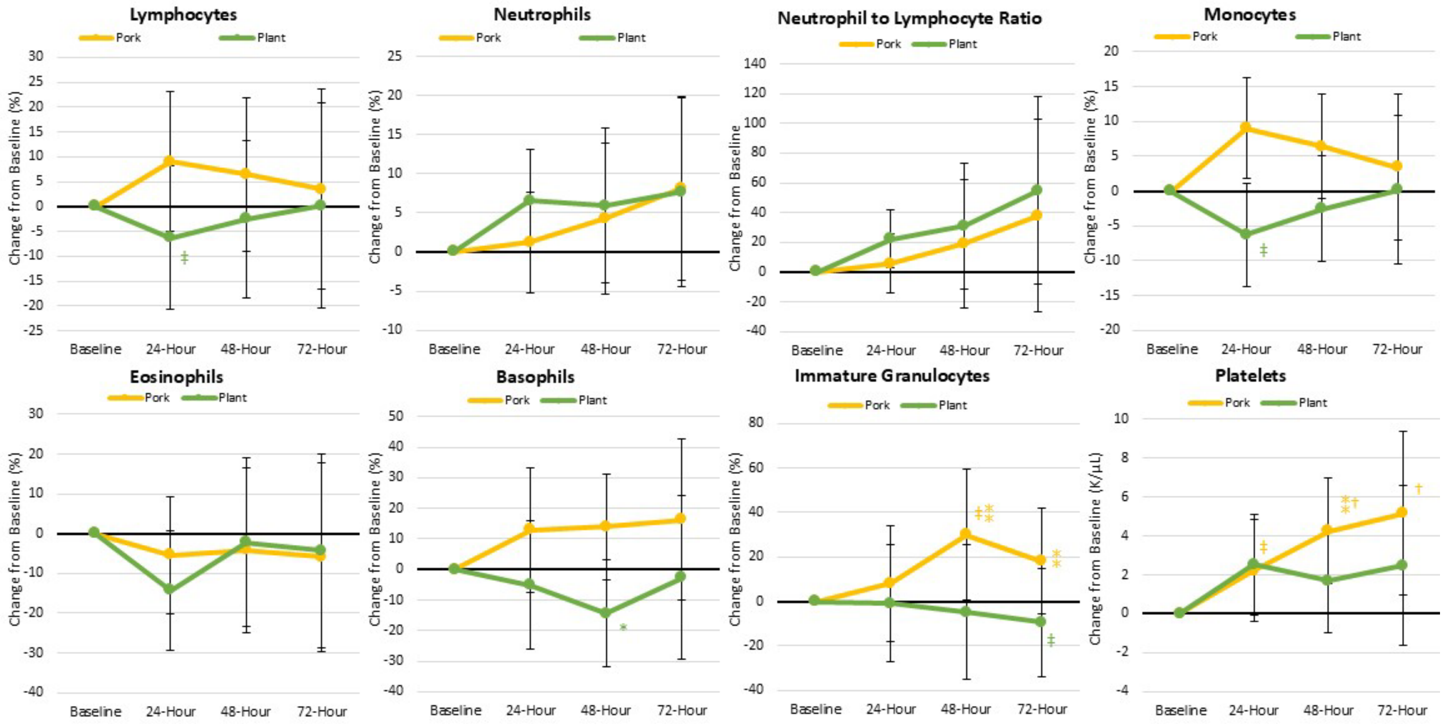

Figure S3b. Percent changes in whole blood white blood cells. Data are displayed as mean percentage changes from baseline with 95% confidence intervals. † = $p < 0.05$ (‡ = $p > 0.05$ to $p < 0.10$) from baseline values. * $p < 0.05$ (⁑ = $p > 0.05$ to $p < 0.10$) difference between diet treatments.

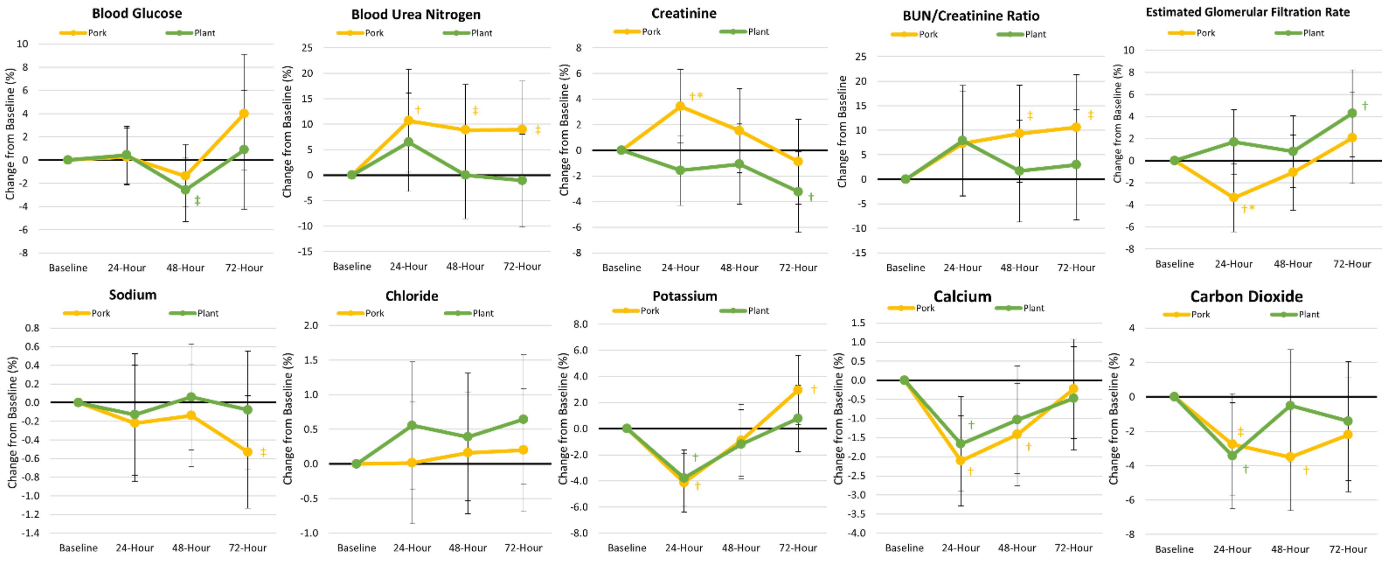

Figure S4. Percent changes renal function and electrolyte-related variables. Data are displayed as mean percentage changes from baseline with 95% confidence intervals. † = $p < 0.05$ (‡ = $p > 0.05$ to $p < 0.10$) from baseline values. * = $p < 0.05$ difference between diet treatments.

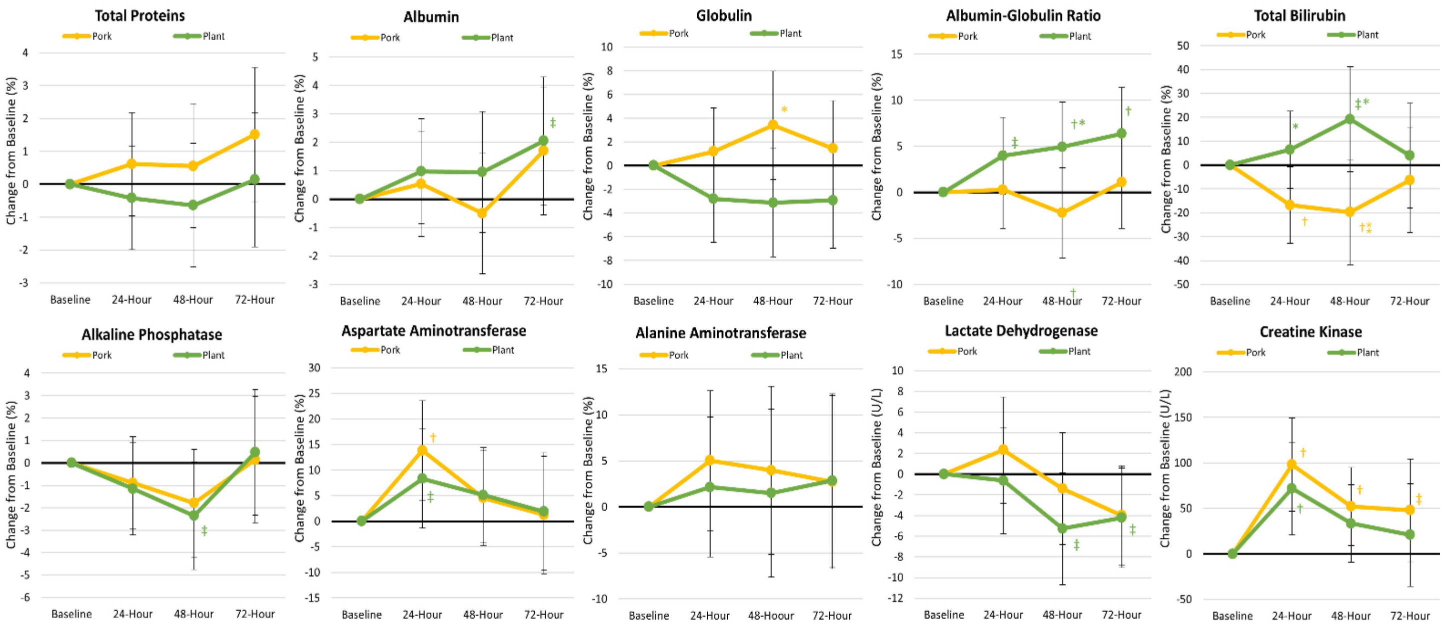

Figure S5. Percent changes in markers of catabolism and enzymes. Data are displayed as mean percentage changes from baseline with 95% confidence intervals. † = $p < 0.05$ (‡ = $p > 0.05$ to $p < 0.10$) from baseline values. * = $p < 0.05$ (⁑ = $p > 0.05$ to $p < 0.10$) difference between diet treatments.

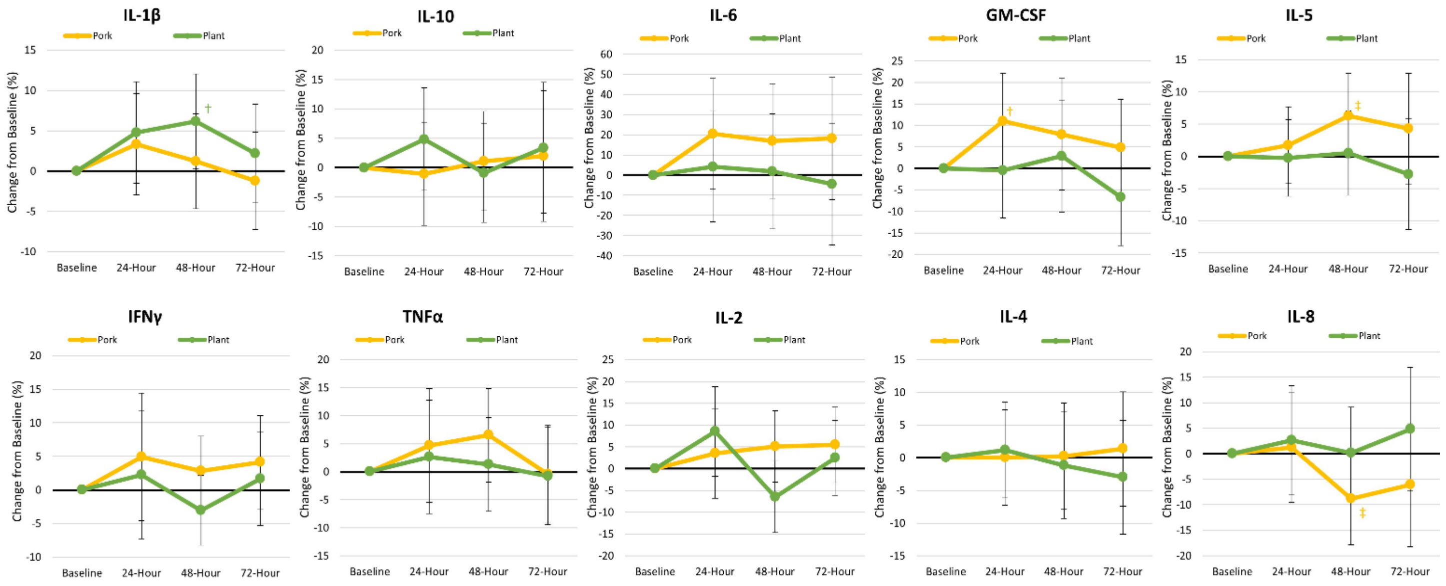

Figure S6. Percent changes in cytokines. Data are displayed as mean percentage changes from baseline with 95% confidence intervals. † = $p < 0.05$ (‡ = $p > 0.05$ to $p < 0.10$) from baseline values.

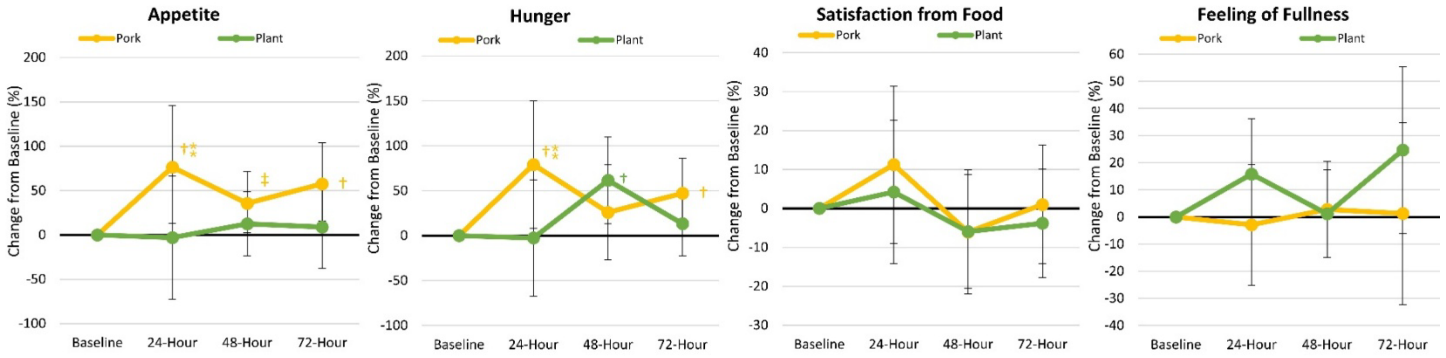

Figure S7. Percent changes in cytokines. Data are displayed as mean percentage changes from baseline with 95% confidence intervals. † = $p < 0.05$ from baseline values. ‡ = $p>0.05$ to $p < 0.10$ from baseline values. ⁑ Represents $p>0.05$ to $p < 0.10$ difference between diet treatments.
